# Evolution of neutrophil extracellular traps in the pathology of stroke

**DOI:** 10.4103/NRR.NRR-D-25-00364

**Published:** 2025-09-03

**Authors:** Wenjing Ning, Qian Wang, Yuzhen Xu

**Affiliations:** 1 Department of Rehabilitation, The Second Affiliated Hospital of Shandong First Medical University, Taian, Shandong Province, China; 2 Department of Central Laboratory, The Affiliated Taian City Central Hospital of Qingdao University, Taian, Shandong Province, China

**Keywords:** atherosclerosis, cerebral hemorrhage, cerebrovascular disorders, intracranial, sinus thrombosis, stroke, subarachnoid hemorrhage, therapeutic, thrombosis, vascular endothelium

## Abstract

Stroke is a major cause of death and disability worldwide, and its pathogenesis is complex, involving multiple pathological processes, such as thrombosis, ischemia-reperfusion injury, inflammatory response, and blood–brain barrier disruption. In recent years, neutrophil extracellular traps have been found to be involved in the body’s anti-infection defense and to play an important role in stroke. Studies have shown that neutrophil extracellular traps promote thrombus expansion and neuroinflammation in ischemic stroke, and they may be involved in disease progression and recovery in hemorrhagic stroke by modulating local inflammation and influencing hematoma clearance. This review systematically summarizes the evolution and mechanism of action of neutrophil extracellular traps in stroke pathology. Reactive oxygen species drive the formation of neutrophil extracellular traps 6–24 hours after cerebral infarction. At 24–48 hours, they exacerbate vascular injury and thrombosis, at 48–72 hours, they aggravate neurological injury, and after 72 hours, neutrophil extracellular traps are involved in the disruption of the blood–brain barrier and the maintenance of the inflammatory response. During stroke development, neutrophil extracellular traps are involved in multiple pathological mechanisms after cerebral infarction. They induce vascular endothelial damage, exacerbating vascular leakage and edema, injuring neurons, inducing apoptosis, promoting thrombosis, participating in reperfusion injury, and damaging the blood–brain barrier. In hemorrhagic stroke, neutrophil extracellular traps are closely associated with hematoma clearance, early brain injury, and delayed cerebral ischemia, and can be used as a biomarker to assess disease progression and efficacy. In the acute phase of stroke, neutrophil extracellular traps mainly promote injury, and in the chronic phase, they mainly promote repair. Neutrophil extracellular traps, as an important biomarker of stroke, are closely correlated with stroke severity. Additionally, neutrophil extracellular traps play an important role in atherosclerosis and intracranial venous thrombosis. Current research has confirmed that deoxyribonuclease is a key drug for degrading neutrophil extracellular traps and has shown significant therapeutic potential. Peptidyl arginine deiminase 4 inhibitors and high mobility group box 1 antagonists effectively inhibit the formation of neutrophil extracellular traps through their own unique mechanisms. Multi-targeted intervention strategies for neutrophil extracellular traps have shown broad clinical application prospects. Neutrophil extracellular traps exhibit synergistic effects with anticoagulants and thrombolytic drugs, and interventions targeting neutrophil extracellular traps can influence the efficacy of anticoagulation and thrombolytic therapy. These findings provide a theoretical basis for developing new anticoagulation and thrombolysis strategies for stroke and improving clinical outcomes for patients.

## Introduction

Stroke is one of the leading causes of death and disability worldwide, mainly comprising ischemic stroke and hemorrhagic stroke (Hilkens et al., 2024). Its pathogenesis is complex, involving thrombus formation, ischemia-reperfusion injury, vascular rupture, secondary inflammatory responses, and blood–brain barrier (BBB) disruption (Li et al., 2025). In recent years, the role of the innate immune system, particularly neutrophil extracellular traps (NETs), in stroke has garnered increasing attention, and NETs are considered a potential critical pathological factor regulating stroke progression.

NETs are net-like structures released by neutrophils, primarily composed of deoxyribonucleic acid (DNA), histones, and antimicrobial proteins. Initially, they were recognized as part of the innate immune defense against pathogen infection. However, subsequent studies revealed that NETs play a dual role in cerebrovascular lesions and the pathological process of stroke. On one hand, they may protect the brain by clearing pathogens and regulating inflammation. On the other hand, excessively or abnormally activated NETs can exacerbate pathological damage in stroke (Sun et al., 2023; Lou et al., 2024; Wu et al., 2024).

In ischemic stroke, NETs affect disease progression by multiple mechanisms, including promoting thrombosis, exacerbating ischemic injury, inducing BBB damage, and enhancing the neuroinflammatory response (Wu et al., 2024). NETs may also aggravate microvascular embolism and endothelial dysfunction in the ischemia-reperfusion injury process, thereby exacerbating neuronal injury (Huang et al., 2024a). In hemorrhagic stroke (including intracerebral hemorrhage and subarachnoid hemorrhage), NETs may play an important role in secondary brain injury by regulating local inflammatory responses and affecting hematoma formation and absorption, thus affecting disease progression and prognosis (Zhou et al., 2023). Therefore, an in-depth investigation of the mechanism of NETs in stroke not only has important pathophysiological significance but also may provide new strategies for early diagnosis and the precise treatment of stroke.

This review summarizes the mechanism of action of NETs in the pathological process of stroke, including their production in cerebral infarction lesions, their relationship with vascular endothelial injury, their influence on nerve cells and blood vessels, their association with thrombosis, and their complex interactions with reperfusion injury and BBB damage. Additionally, this paper explores the potential value of NETs as a biomarker for stroke and cerebral infarction, summarizes therapeutic strategies targeting NETs, and discusses the mechanisms of action and clinical significance of NETs in hemorrhagic stroke. It also comprehensively examines targeted therapeutic strategies for NETs, such as DNase-mediated degradation, peptidyl arginine deiminase 4 inhibitors, and NET-targeted anti-inflammatory interventions. Finally, we highlight future research directions. The aim of this article is to emphasize that precise modulation of NETs may provide new strategies for stroke treatment and contribute to improved clinical outcomes for patients.

Studies on the direct association between NETs and nerve regeneration have remained relatively limited in recent years. However, increasing evidence suggests that the neuroinflammatory microenvironment mediated by NETs may considerably affect subsequent neural regeneration (Au and Ma, 2022; Sha et al., 2023). NETs not only exacerbate ischemic neuronal death by releasing toxic components such as histones and proteases (Lee et al., 2017), but they may also persistently activate microglial cells (Au and Ma, 2022) and disrupt the integrity of the BBB (Haruwaka et al., 2019; Chou et al., 2023). Through these mechanisms, NETs may inhibit endogenous neural stem cell proliferation and synaptic remodeling (Zhou et al., 2020b; Au and Ma, 2022), ultimately affecting neural regeneration. While this review focuses primarily on the close relationship between NETs and neuroinflammation, thrombosis, neurons, and vasculature, it also provides a theoretical basis for neuroregeneration research and identifies a new target for the development of synergistic immune-modulation and neuroregeneration therapies. By targeting NETs, we aim to reduce acute brain injury and create a favorable microenvironment for neural repair, a dual benefit that could represent an important breakthrough in stroke treatment.

The aim of this review is to comprehensively explore the mechanisms of NET formation in stroke, its clinical significance, and its potential value as a therapeutic target. We discuss the generation of NETs in cerebral infarction, their relationship with vascular endothelial injury, their effects on neuronal cells and blood vessels, their association with thrombosis, and the complex interactions with reperfusion injury and BBB injury from the perspective of underlying pathological mechanisms. Additionally, we analyze the potential of NETs as biomarkers of cerebral infarction, as well as the mechanisms of action and clinical significance of NETs in hemorrhagic stroke, and we comprehensively explore targeted therapeutic strategies against NETs. By summarizing existing studies and looking toward future directions, this article aims to provide new approaches to mechanistic research and clinical treatment of stroke.

## Search Strategy

A literature search was conducted in the PubMed database using the following keywords: “Atherosclerosis,” “Hemorrhagic stroke,” “Cerebral hemorrhage,” “Cerebrovascular disorders,” “Ischemic stroke,” “Neutrophil extracellular traps (NETs),” “Nervous system injuries,” “Neuroinflammatory disease,” “Reperfusion injury,” “Stroke,” “Subarachnoid hemorrhage,” “Sinus thrombosis,” “Therapeutic,” and “Vascular endothelium.” Various combinations of these keywords were used to obtain comprehensive and high-quality studies. The search period was limited to publications in English from January 2000 to January 2025.

During the preliminary screening, irrelevant studies were excluded based on the relevance of their titles and abstracts. Full-text analysis was performed for studies that met the research objectives. Key information extracted included the mechanisms of NET formation, the role of NETs in stroke progression, their potential as biomarkers, and therapeutic strategies targeting NETs. The selected studies were categorized based on their effects on pathophysiology, clinical significance, and therapeutic value. A total of 273 documents were ultimately included in the review analysis. The literature review encompassed both animal experiments (primarily rodent models) and clinical studies involving stroke patients, with over 90% of the cited references published between 2018 and 2025 to ensure the timeliness and relevance of the content.

## Stroke

### Ischemic stroke

Cerebral infarction is one of the leading causes of death and disability worldwide (Liang et al., 2025; Shen et al., 2025). Its pathomechanism is complex and primarily involves a series of interrelated processes, including vascular occlusion, ischemia-reperfusion injury, inflammatory response, and neuronal cell death (Boursin et al., 2018; Hu et al., 2025).

Cerebral infarction is usually caused by a thrombus or embolism, leading to the occlusion of cerebral arteries, resulting in severe ischemia and insufficient oxygen delivery in the blood supplied to the neural tissues in the area. Localized damage to the vascular endothelium and the rapid accumulation of platelets and clotting factors promote thrombosis, further exacerbating blood flow interruption (Tater and Pandey, 2021).

After cerebral infarction, the local inflammatory response is rapidly initiated and many immune cells, such as neutrophils, macrophages, and lymphocytes, are recruited to the infarcted area (Potter et al., 2022). Neutrophils not only exacerbate tissue damage by releasing inflammatory mediators, but also promote thrombosis and a local inflammatory response by releasing NETs, which play an important role in the progression of cerebral infarction (Kang et al., 2020).

The disruption of oxygen and nutrient supply because of ischemia causes neurons to rapidly enter a state of energy depletion, which, in turn, leads to necrosis and apoptosis. Intracellular calcium overload, glutamate toxicity, and oxidative stress combine to accelerate neuronal death, leading to irreversible neurological damage and dysfunction (Orellana-Urzúa et al., 2020).

**Figure 1:**
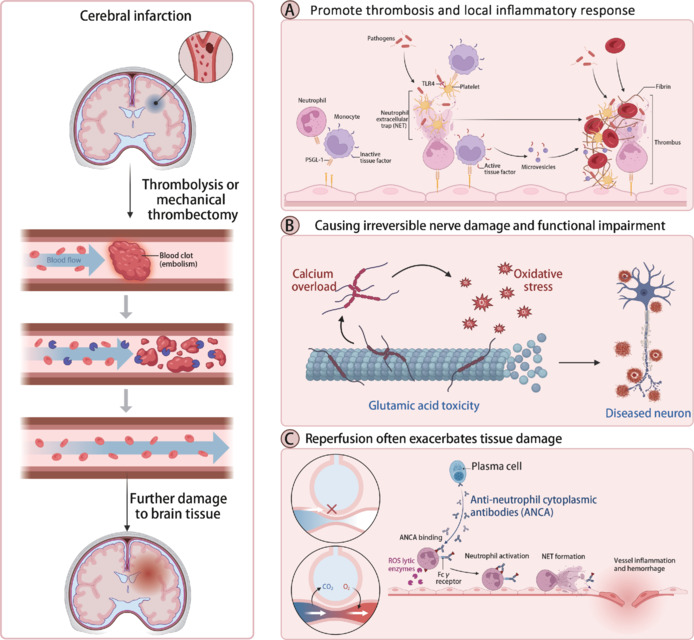
Mechanism of cerebral infarction. (A) Cerebral infarction leads to thrombus formation. (B) Cerebral infarction results in neuronal cell death. (C) Brain tissue is further damaged following ischemia-reperfusion. ANCA: Anti-neutrophil cytoplasmic antibodies; NET: neutrophil extracellular trap; PSGL-1: P-selectin glycoprotein ligand-1; TLR4: Toll-like receptor 4.

Although treatments, such as thrombolysis and mechanical retrieval, can partially restore blood flow, the oxidative stress and inflammatory response associated with reperfusion tend to exacerbate tissue damage. After reperfusion, excessive reactive oxygen species (ROS) are produced, inducing apoptosis and necrosis and releasing large amounts of inflammatory mediators, leading to further damage to brain tissue (Potter et al., 2022; **Figure 1**).

### Hemorrhagic stroke

Hemorrhagic stroke is a severe cerebrovascular event caused by the rupture of cerebral blood vessels, leading to the entry of blood into the brain parenchyma or cerebrospinal fluid circulation system. It primarily includes intracerebral hemorrhage, subarachnoid hemorrhage, and often trauma-induced subdural hemorrhage and epidural hemorrhage (Hilkens et al., 2024). Its pathological mechanisms are complex and involve multiple factors, including abnormalities in vessel wall structures and reduced resistance to mechanical stress, hemodynamic changes, imbalance between coagulation and fibrinolysis systems, and inflammatory responses (Li et al., 2025).

The most common cause of intracerebral hemorrhage is hypertensive small vessel disease. Chronic hypertension can lead to hyaline degeneration in small arteries, lipid infiltration, and smooth muscle cell necrosis, making the vessel walls fragile and increasing the risk of hemorrhage. Intracerebral hemorrhage is also seen in cerebral amyloid angiopathy, where amyloid-β protein deposits in the cortical and leptomeningeal vessels, weakening vessel wall integrity and making them prone to cortical and subcortical hemorrhage. The rupture of an intracranial aneurysm and arteriovenous malformations are the main causes of subarachnoid hemorrhage (Yakhkind et al., 2025).

After vascular rupture and hemorrhage, hematoma formation and expansion are regulated by coagulation and fibrinolytic systems, and coagulation-fibrinolytic imbalance can affect the severity of hemorrhagic stroke. After hemorrhage, the mechanical compression of the hematoma, the toxic effects of erythrocyte lysis products, and the release of inflammatory mediators combine to induce secondary brain injury. Hematoma disintegration releases hemoglobin, heme, and ferric ions (Fe^2+^), which can trigger oxidative stress, inducing neuronal damage and BBB disruption. Meanwhile, the rapid aggregation of neutrophils and release of NETs, and oxygen free radicals may aggravate brain tissue injury around the hematoma. M1-type microglia release pro-inflammatory cytokines [e.g., interleukin-1β (IL-β) and tumor necrosis factor-α (TNF-α)], which exacerbate inflammation and BBB damage, while M2-type microglial cells may promote repair and hematoma resorption (Ning et al., 2025). Vascular rupture and inflammation-mediated endothelial injury can lead to increased BBB permeability and plasma protein leakage, triggering vasogenic brain edema and further exacerbating intracranial hypertension. Brain tissue injury may release tissue factor and initiate exogenous coagulation pathways, while the activation of the fibrinolytic system after hematoma formation may lead to a secondary hemorrhage (Seiffge et al., 2024).

Recent studies have shown that NETs play a crucial role in the progression of hemorrhagic stroke (Zhao et al., 2023b; Zhou et al., 2023). Histones and proteases released by NETs can exacerbate BBB disruption and brain tissue damage. NETs act as a scaffold for thrombus formation, promoting local coagulation. They may also cause a secondary hemorrhage via the fibrinolysis system. The continuous release of NETs may obstruct hematoma clearance by forming dense DNA fibers and inducing chronic inflammatory responses (Zhou et al., 2023).

## Neutrophil Extracellular Traps

Neutrophils are the most abundant white blood cells in the circulatory system and serve as the first line of defense in response to infection or injury (Liew and Kubes, 2019). They perform a broad range of functions, including microbial phagocytosis, the release of pro-inflammatory cytokines and chemokines, oxidative bursts, and the formation of NETs (Chakraborty et al., 2023).

NETs were first discovered by Brinkmann et al. in 2004. When neutrophils are stimulated by specific triggers (such as bacteria, viruses, cytokines, or ROS), their nuclear membranes rupture, releasing chromatin that binds to cytoplasmic proteins and forms fibrous, web-like structures in the extracellular space. This formation is accompanied by neutrophil death, representing a unique form of cell death distinct from apoptosis and necrosis, termed NET-associated cell death (NETosis) (Thiam et al., 2020). Structurally, NETs primarily consist of DNA, histones, and various granule-associated proteins, including elastase, myeloperoxidase (MPO), and calprotectin (Xu et al., 2022; Zhang et al., 2023a).

The formation of NETs is an important neutrophil effector function. NETs not only play an important role in anti-infective immunity, but evidence indicates that NETs are involved in the development of neurological disorders, such as Alzheimer’s disease (Zenaro et al., 2015; Shao et al., 2024), multiple sclerosis (Papayannopoulos, 2018; De Bondt et al., 2020), amyotrophic lateral sclerosis (Murdock et al., 2021), and Parkinson’s disease (Wang et al., 2024b). NETs have been associated with retinal vascular remodeling (Binet et al., 2020), stroke (Kang et al., 2020), and traumatic brain injury (Vaibhav et al., 2020). Other studies found that NETs play an important role in the formation of acute cerebral infarction (Sun et al., 2023; Zeineddine et al., 2024), and through their interactions with the coagulation system, NETs can accelerate thrombus formation and exacerbate neurological damage (Tater and Pandey, 2021).

**Figure 2:**
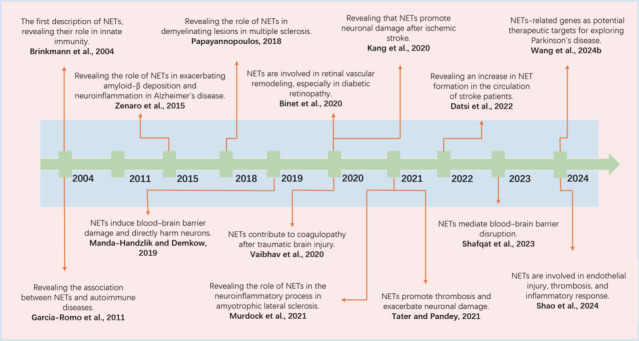
Research progress of NETs in neurological diseases. This timeline systematizes the key milestones in the study of NETs (2004–2024), covering their key roles in innate immunity, thrombosis, neurodegenerative diseases (e.g., Alzheimer’s disease and Parkinson’s disease), autoimmune diseases, and cerebrovascular diseases (e.g., stroke and traumatic brain injury). Studies have shown that NETs exacerbate the pathology of various diseases by mediating inflammatory responses, blood–brain barrier disruption, and neurological damage, and have recently emerged as a potential therapeutic target for neuroimmunomodulation. Each entry is labeled with the key authors and year, reflecting the chronology and reliability of the research evidence. NETs: Neutrophil extracellular traps.

NETs also play an important role in the pathological process of hemorrhagic stroke (Dinc and Ardic, 2025), and were found to further aggravate secondary brain injury by promoting a perihematoma inflammatory response, exacerbating BBB disruption, and affecting hematoma clearance (Zhang et al., 2024). At the same time, inflammatory mediators and protein hydrolases released by NETs may affect vascular integrity, which may increase the risk of a secondary hemorrhage. NETs are involved in the pathological processes of endothelial injury, thrombosis, and the inflammatory response. Thus, their different mechanisms of action in ischemic and hemorrhagic strokes are worthy of further in-depth study (Shao et al., 2024; **Figure 2**).

## Neutrophil Extracellular Traps and Ischemic Stroke

### Emergence of neutrophil extracellular traps

When cerebral infarction occurs, ischemic injury activates the inflammatory response, and many neutrophils rapidly accumulate at the site of injury and infiltrate the central nervous system (CNS) (Otxoa-de-Amezaga et al., 2019). In one experiment, neutrophils were detected in soft meninges, ventricles, capillary lumens, perivascular spaces, and parenchyma within the infarcted core of cerebral ischemia-reperfusion mice (Otxoa-de-Amezaga et al., 2019). The number of neutrophils increased over time and was positively correlated with neuronal loss, infarct size, and cognitive dysfunction. This experiment confirms neutrophil degranulation and the formation of NETs during ischemia.

At 0 to 6 hours after the onset of cerebral infarction, cerebral ischemia leads to local hypoxia, energy depletion, and necrosis of neuronal cells. This triggers irreversible necrosis in the core, as well as dysfunction and inflammatory responses in the ischemic penumbra (Feske, 2021). Cellular necrosis releases damage-associated molecular patterns (DAMPs), such as HMGB1, adenosine triphosphate (ATP), and heat shock proteins. DAMPs activate endothelial and immune cells through pattern recognition receptors (e.g., Toll-like receptors 2 (TLR2) and 4 (TLR4)) to initiate inflammatory responses (Tuo et al., 2022). ATP exerts pro-inflammatory effects on immune cells, such as microglia and monocytes (Li et al., 2022), through P2X7 receptors (Ning et al., 2025).

Activated endothelial cells and neurons release chemokines (e.g., IL-8, C–X–C motif chemokine ligand 1, C–X–C motif chemokine ligand 2) and cytokines (e.g., TNF-α and IL-1β; Li et al., 2022). Localized hypoxia activates the hypoxia-inducible factor-α (HIF-α) pathway. HIF-α stabilization not only promotes vascular endothelial growth factor-mediated vascular responses but also enhances the activity of nuclear factor κ-light-chain-enhancer of activated B cells (NF-κB), which regulates chemokine expression and enhances neutrophil recruitment. This drives neutrophil migration from the circulation to the ischemic region (Cui et al., 2021).

Neutrophils cross the BBB into the brain parenchyma by binding to adhesion molecules (e.g., intercellular adhesion molecule 1 and vascular cell adhesion molecule 1) on endothelial cells via integrins (e.g., lymphocyte function-associated antigen 1 and macrophage-1 antigen) on their surface (Kang et al., 2020). Peripheral blood neutrophil counts in stroke mice were reported to peak 12 hours after cerebral ischemia, followed by a secondary peak at the site of brain injury occurring 1 to 2 days later (Cai et al., 2020). Additionally, the number of neutrophils in a patient’s peripheral blood increases rapidly after the onset of a stroke, and higher neutrophil counts correlate with a poor stroke prognosis (Cai et al., 2020).

Between 6 and 24 hours after cerebral infarction, activated neutrophils generate large amounts of ROS through nicotinamide adenine dinucleotide phosphate oxidase, which serves as a key driving force for NET formation (Vorobjeva and Chernyak, 2020). Activated HIF-α enhances NF-κB activity, indirectly regulating the function of the nicotinamide adenine dinucleotide phosphate oxidase complex and promoting ROS production (Cui et al., 2021). ROS activates downstream signaling pathways, including mitogen-activated protein kinase and NF-κB, further amplifying the inflammatory response (Liu et al., 2024). Additionally, ROS activate peptidyl arginine deiminase 4 (Liu et al., 2024), which catalyzes the citrullination of arginine residues in histones 3 and 4, leading to chromatin decondensation. The decondensed chromatin binds to granule proteins, such as MPO, neutrophil elastase, and cathepsin G, forming NETs, which are subsequently released extracellularly (Yadav et al., 2019). NETs are primarily detected between the second and third days following a stroke (Cai et al., 2020). Studies have confirmed that circulating neutrophils from ischemic stroke patients exhibit higher NET formation compared to those from healthy controls (Denorme et al., 2021; Datsi et al., 2022).

At 24–48 hours after the onset of cerebral infarction, histones (e.g., H3 and H4), granule proteins (e.g., MPO and neutrophil elastase), and extracellular DNA in NETs activate endothelial cells, which directly injure the endothelial barrier and disrupt vascular integrity (Zhang et al., 2023a). DNA in NETs activates the endogenous coagulation cascade through FXII (Burmeister et al., 2022), whereas histones promote platelet aggregation through the TLR4/protease-activated receptor 4 axis and aggravate microvascular thrombosis, leading to apoptosis and BBB damage (Kim et al., 2021). Histones activate the NF-κB pathway by binding to TLR2 and TLR4 on the surface of endothelial cells, inducing the release of inflammatory factors (e.g., interleukin-6 and interleukin-8). The TLR4/MYD88/NF-κB pathway promotes the expression of cellular adhesion molecules (ICAM-1 and E-selectin) to increase leukocyte adhesion and infiltration (Kim et al., 2021). The extracellular in NETs is recognized by endothelial cyclic GMP-AMP synthase (cGAS), which activates stimulator of interferon genes (STING) signaling and induces type I interferon (IFN-α/β) release, further exacerbating inflammation (Zhao et al., 2023a).

At 48–72 hours after the onset of cerebral infarction, MPO and NE released from NETs can induce oxidative stress and inflammatory cascade responses, synergizing with matrix metalloproteinases (MMPs) to degrade the neuronal extracellular matrix, which indirectly leads to neuronal injury (Tembhre et al., 2022). Histones (H3 and H4) activate NF-κB and c-Jun N-terminal kinase signaling via TLRs and the receptor for advanced glycation end products (RAGE), enhancing neuronal inflammatory responses and indirectly leading to mitochondrial dysfunction and apoptosis (Anfinogenova et al., 2020). Damaged neurons release DAMPs (e.g., HMGB1 and S100 calcium-binding protein B), which further activate neutrophils and microglia, creating a vicious cycle (Ning et al., 2025).

At 72 hours after cerebral infarction, NETs can upregulate the expression of MMP-9 through inflammatory factors (IL-1β and TNF-α). MMP-9 degrades zonula occludens-1 and occludin, destroys the BBB, and increases edema and plasma protein exudation (Cheung et al., 2024). NETs initiate an inflammatory cascade and further enhance BBB permeability (Huang et al., 2022). After BBB disruption, C–X–C motif chemokine ligand 12 and C–X–C chemokine receptor type 4 signaling promotes monocyte and T cell infiltration and exacerbates the inflammatory response and tissue damage (Chou et al., 2023).

In ischemic stroke, the formation of NETs is a multistep process driven by ROS, peptidyl arginine deiminase 4, and histone citrullination. NETs exacerbate inflammation, thrombosis, and tissue injury through interactions with endothelial cells, neurons, and the BBB (**Box 1**).

**Box 1:** Pathological mechanisms at different time stages of ischemic stroke and the role of NETs

**0–6 h: Acute ischemia and initial inflammatory response**
(1) Local hypoxia and energy depletion → Neuronal necrosis (Feske, 2021)
Core region: Irreversible necrosis
Ischemic penumbra: Functional impairment and inflammation
(2) Release of DAMPs (HMGB1, ATP, HSPs) (Tuo et al., 2022)
TLR2/TLR4 → Activation of endothelial cells and immune cells → Inflammatory response
ATP-P2X7 → Pro-inflammatory effects (microglia and monocytes)
(3) Release of pro-inflammatory factors (Li et al., 2022)
Chemokines (IL-8, CXCL1, and CXCL2)
Cytokines (TNF-α and IL-1β)
(4) Activation of the HIF-α pathway (Cui et al., 2021)
HIF-1α stabilization → VEGF-mediated vascular response & enhanced NF-κB activity
NF-κB → Upregulation of chemokines → Increased neutrophil recruitment
(5) Neutrophil migration (Cai et al., 2020)
LFA-1/Mac-1 ↔ ICAM-1/VCAM-1 →BBB penetration
**6–24 h: ROS-driven NET formation**
(1) NADPH oxidase (NOX2) → Excessive ROS production (driving force of NET formation) (Vorobjeva and Chernyak, 2020)
HIF-α → NF-κB → Enhancement of NADPH oxidase complex function
ROS → MAPK/NF-κB pathway → Amplification of inflammation
(2) PAD4 activation (Liu et al., 2024)
Citrullination of histones H3/H4 → Chromatin decondensation
Chromatin + MPO/NE/Cathepsin G → NET formation
(3) NET release (detectable on days 2–3 post-stroke) (Yadav et al., 2019; Denorme et al., 2021; Datsi et al., 2022)
Elevated NET formation in circulating neutrophils of stroke patients
**24–48 h: NETs exacerbate vascular injury and thrombosis**
(1) Endothelial damage (Zhang et al., 2023a)
NET-associated histones (H3/H4), MPO, NE, and DNA → Vascular integrity disruption
FXII → Intrinsic coagulation cascade activation
TLR4/PAR4 → Platelet aggregation → Microvascular thrombosis
(2) Inflammatory amplification (Kim et al., 2021; Burmeister et al., 2022)
TLR4/MYD88/NF-κB → ↑ IL-6, IL-8 → Pro-inflammatory response
ICAM-1, E-selectin↑ → Leukocyte infiltration
(3) STING pathway activation (cGAS recognition of extracellular DNA) (Zhao et al., 2023a)
IFN-α/β ↑ → Exacerbation of inflammation
**48–72 h: NETs aggravate neuronal injury**
(1) Oxidative stress and extracellular matrix degradation (Tembhre et al., 2022)
MPO/NE + MMPs → Breakdown of neuronal extracellular matrix
(2) Histones H3/H4 → TLRs/RAGE activation (Anfinogenova et al., 2020)
NF-κB/JNK signaling → Neuroinflammation & mitochondrial dysfunction → Neuronal apoptosis
(3) DAMP-mediated cyclic activation (HMGB1 and S100B) (Ning et al., 2025)
Enhanced activation of neutrophils & microglia → Vicious inflammatory cycle
**After 72 h: BBB disruption and sustained inflammation**
(1) MMP-9↑(Huang et al., 2022; Cheung et al., 2024)
Degradation of ZO-1/Occludin →BBB breakdown → Increased edema
(2) CXCL12-CXCR4 signaling activation (Chou et al., 2023)
Monocyte and T-cell infiltration → Worsening tissue damage

Here systematically summarizes the key pathophysiological processes that occur 0–72 h after the onset of acute ischemic stroke, with a focus on revealing the central role of NETs in disease progression. Studies are divided into four phases according to the timeline: acute ischemia (0–6 h), NET formation (6–24 h), vascular injury (24–48 h), and neuronal injury (after 48–72 h). Each phase is elucidated in detail, highlighting the molecular mechanisms and cellular events involved. This summary provides an important theoretical basis for stroke therapy, suggesting NETs as novel therapeutic targets and laying the groundwork for the development of time window-specific therapies (e.g., targeting PAD4, NOX2, or NET-component neutralization strategies). These findings not only deepen the understanding of the pathological mechanisms of stroke but also offer potential intervention strategies to improve clinical outcomes. BBB: Blood–brain barrier; CXCL1: C–X–C motif chemokine ligand 1; CXCL2: C–X–C motif chemokine ligand 2; CXCL12: C–X–C motif chemokine ligand 12; DAMPs: damage-associated molecular patterns; FXII: coagulation factor XII; HIF-α: hypoxia-inducible factor alpha; HMGB1: high mobility group box 1; HSPs: seat shock proteins; ICAM-1: intercellular adhesion molecule 1; IFN-α/β: interferon-alpha/beta; IL-1β: interleukin-1 beta; IL-6: interleukin-6; IL-8: interleukin-8; JNK: c-Jun N-terminal kinase; LFA-1: lymphocyte function-associated antigen 1; Mac-1: macrophage-1 antigen; MAPK: mitogen-activated protein kinase; MMPs: matrix metalloproteinases; MMP-9: matrix metalloproteinase-9; MPO: myeloperoxidase; MYD88: myeloid differentiation primary response 88; NADPH: nicotinamide adenine dinucleotide phosphate; NE: neutrophil elastase; NETs: neutrophil extracellular traps; NF-κB: nuclear factor-kappa beta; NOX2: NADPH oxidase 2; PAD4: peptidyl arginine deiminase 4; PAR4: protease-activated receptor 4; P2X7: P2X purinoceptor 7; RAGE: receptor for advanced glycation end products; ROS: reactive oxygen species; S100B: S100 calcium-binding protein B; STING: stimulator of interferon genes; TLR2: Toll-like receptor 2; TLR4: Toll-like receptor 4; TNF-α: tumor necrosis factor-alpha; VCAM-1: vascular cell adhesion molecule 1; VEGF: vascular endothelial growth factor; ZO-1: zonula occludens-1.

A study by van de Graaf et al. (2018) analyzed thrombi retrieved through catheter therapy from 108 patients with acute AIS. The histological findings revealed that NETs play a role in the composition of all AIS thrombi, notably in the outer layers. A study involving 37 human thromboemboli from patients with ischemic stroke undergoing mechanical thrombectomy found that neutrophils were the main cellular component of cerebral thromboemboli, although significant morphological heterogeneity was observed (Essig et al., 2020). Neutrophils accumulate at the peripheral regions of fibrin-rich structures, suggesting that they may interact with different structural components of the thrombus. NETs were detected in all 35 thromboemboli examined, in varying quantities. NETs were almost exclusively located in fibrin-rich areas. Neutrophils and NETs are key components of cerebral thromboemboli and contribute to the complexity of their structure.

The generation of NETs is associated with several key biomarkers, including extracellular DNA released into the surrounding environment, proteins derived from neutrophils (e.g., MPO and neutrophil elastase), and histone H3 that has undergone citrullination (H3Cit). Additionally, essential components such as peptidyl arginine deiminase 4 and citrullinated histones, along with their molecular complexes, play critical roles in this process (Zhao et al., 2023b).

Shortly after permanent middle cerebral artery occlusion (MCAO) in rats, H3Cit (a marker of NETosis) was induced in neutrophils in the meninges and peripheral blood. H3Cit-positive cells entered the brain tissue through the meninges 6 hours post-MCAO and were subsequently observed in the cerebral cortex at 12 hours, followed by the striatum. Notably, the induction of H3Cit begins in circulating neutrophils, which then migrate into the brain parenchyma, and these cells may be either intact or dissolved (Kim et al., 2019). The co-localization of carcinoembryonic antigen-related cell adhesion molecule 8 or cluster of differentiation 66b, DNA, H3Cit, neutrophil elastase, citrullinated histone H4, MPO, and DNA in the thrombi of AIS patients revealed the abundant presence of neutrophils and NETs (Luo et al., 2023).

Platelet-derived HMGB1 is a major inducer of NET formation in ischemic stroke, with platelets identified as the critical source of HMGB1 responsible for the formation of NETs during the acute phase of stroke. After a stroke, platelet depletion or the specific knockout of HMGB1 was reported to significantly reduce the levels of HMGB1 and NETs in plasma, greatly improving stroke prognosis (Denorme et al., 2022). Treatment with a novel neuroinhibitory factor in mice specifically blocked NET formation, reduced infarct size, and improved long-term neurological and motor function (Denorme et al., 2022). These findings support the significant pathological role of NETs in ischemic stroke and the need for further research into novel neuroinhibitory factors as a therapeutic strategy for stroke.

### Neutrophil extracellular traps and endothelial injury

Vascular endothelial injury triggered by cerebral infarction is closely related to NET production. Endothelial injury exacerbates the inflammatory response of the vessel wall and promotes the crossing of neutrophils through the endothelial barrier into the infarcted area. P-selectin and E-selectin on the surface of endothelial cells further promote the adhesion of neutrophils to the vessel wall and the release of NETs by interacting with receptors on the surface of neutrophils. The release of large amounts of inflammatory factors from damaged endothelial cells is a major driver of neutrophil activation.

The study by Zhou et al. (2020a) examined the levels of NETs, activated platelets, and platelet-derived microparticles in the plasma of 55 stroke patients and 35 healthy controls, suggesting that the levels were significantly higher in plasma from carotid lesion sites compared with aortic arterial blood. Activated platelets in patients with NETs in thrombosis and internal carotid artery occlusion plasma were found to be decorated with phosphatidylserine. Notably, both plasma and thrombin-activated platelets at the site of carotid artery disease are required for NET formation and subsequent phosphatidylserine exposure. Phosphatidylserine-carrying NETs provide a functional platform for the deposition of platelet-derived particles and coagulation factors, thereby increasing thrombin and fibrin formation. DNase I and lactate dehydrogenase significantly inhibited these effects. Additionally, NETs are cytotoxic to endothelial cells, converting these cells to a procoagulant phenotype. This cytotoxicity was blocked by 25%, 39%, or 52% sivelestat, anti-MMP-9, and activated protein C, respectively. NETs play a critical role in the hypercoagulable state of stroke patients, and strategies to prevent the formation of NETs may offer potential therapeutic approaches for thromboembolic intervention (Zhou et al., 2020a).

After traumatic brain injury, inflammatory responses induce the upregulation of chemokines and adhesion molecules within cerebral blood vessels. These signaling molecules play a critical role in attracting neutrophils and facilitating their attachment to the vascular endothelium while also stimulating the release of NETs (Smyth et al., 2018; Jansson et al., 2021). Concurrently, the degradation of the glycocalyx, a proteoglycan-rich layer that normally inhibits interactions between surface molecules, including cell adhesion molecules, enhances neutrophil adhesion to vessel walls (Arts et al., 2021). Once adhered to the vasculature, interactions at the intimal projections, which involve multiple adhesion molecules, further promote the migration of immune cells into the brain parenchyma (Smyth et al., 2022).

A study by Zhang et al. (2021a) found that the levels of NET biomarkers were significantly higher in symptomatic patients with carotid artery stenosis compared to asymptomatic patients and healthy individuals. Elevated levels of neutrophil-platelet aggregates in symptomatic carotid artery stenosis patients induced the generation of NETs. These NETs contributed to the progression of carotid artery stenosis through the action of tissue factors. Additionally, NETs disrupted the endothelial barrier and transformed endothelial cells into a procoagulant phenotype, thereby enhancing the procoagulant state in patients with carotid artery stenosis. Consequently, inhibiting NETs may serve as a potential biomarker and therapeutic target for recurrent stroke in patients with severe carotid artery stenosis (Zhang et al., 2021a).

NETs and neuronal cold-inducible RNA-binding protein (CIRP) expression were found to increase six hours after temporary middle cerebral artery occlusion (tMCAO) and increased significantly after 24 hours, peaking at three days (Li et al., 2024d). NET degradation or CIRP inhibition mitigated cerebral endothelial barrier leakage and reversed decreases in the expression of tight junction proteins (zonula occludens-1, claudin-5, and occludin) in tMCAO mice. *In vitro*, oxygen-glucose-deficient/reperfusion-treated primary neurons or recombinant CIRP induced NET formation through the TLR4/p38 signaling pathway. Transcription factor-specific protein 1 was responsible for the increased expression of CIRP, and the inhibition of specific protein 1 suppressed increases in its expression, decreased NET formation, and reduced brain endothelial barrier leakage in tMCAO mice. Upregulated CIRP levels were also associated with severe brain edema in patients with acute ischemic stroke. After ischemic stroke, the increased expression of transcription factor-specific protein 1 can lead to elevated neuronal CIRP expression and release, which interacts with neutrophils and promotes NET formation, ultimately leading to brain endothelial barrier disruption and edema (Li et al., 2024d).

### Neutrophil extracellular traps and neurovascular injury

NETs exacerbate ischemic brain injury in cerebral infarction by promoting thrombosis through the endothelial lining and directly damaging neurons and blood vessels. The DNA, histones, and proteases (such as elastase and MPO) contained in NETs are cytotoxic and possess strong pro-inflammatory and procoagulant properties, which can directly damage neurons and induce apoptosis. Histones and MPO also intensify the oxidative stress response in the vascular endothelium, induce vascular wall damage, activate platelets and the coagulation cascade, worsen vascular leakage and edema, and aggravate local ischemia, thereby increasing the infarct area.

HMGB1, a DAMP, extensively accumulates in the serum following permanent MCAO and plays a key role in neutrophils both in the brain parenchyma and peripheral blood (Jin et al., 2023b). HMGB1 induces H3Cit through its specific receptors C-X-C chemokine receptor type 4 and TLR4, leading to NETosis. However, it is also released as part of NETs, contributing to neuronal death mediated by NETosis. Thus, a detrimental feedback loop is established between neuronal cell death and NETosis, with HMGB1 serving as a key mediator driving this pathological cycle. In MCAO animal models, the administration of PAD inhibitors to suppress NETosis significantly delayed immune cell infiltration and notably reduced vascular injury (Kim et al., 2019). These findings indicate that HMGB1-induced NETosis amplifies the inflammatory cascade and worsens tissue damage following ischemic brain injury.

The inflammasome, an innate immune complex, activates interleukin IL-18 and IL-1β via caspase-1, and a study using electron microscopy and immunofluorescence showed that NETs are present in thrombi from patients with AIS (Kim et al., 2020a). In addition, the inflammatory vesicle signaling protein caspase-1 and apoptosis-associated speckled proteins containing the caspase recruitment structural domain (ASC) were also present in clots associated with the NETosis marker H3Cit (Kerr et al., 2018). Analysis using a simple protein assay showed significantly higher levels of caspase-1, ASC, and IL-1β, and lower levels of IL-18 in clots compared with plasma from AIS patients and healthy controls. Multivariate analysis showed that IL-1β levels in clots correlated with the number of maneuvers required to achieve complete recanalization, and that ASC, caspase-1, and IL-18 were significant factors affecting recanalization time. Thus, inflammatory vesicle proteins are elevated in NETs in thrombi from patients with AIS, which is associated with poor outcomes after stroke (Chen et al., 2020).

Neovascularization and vascular remodeling have important functions in brain repair after stroke (Yang and Torbey, 2020; Grinchevskaya et al., 2024). A study by Dhanesha et al. demonstrated that neutrophils accumulate in the peripheral cortex of the infarcted area at all stages of ischemic stroke (Dhanesha et al., 2022). Neutrophil-generated intravascular and intracerebral parenchymal NETs peaked on 3 to 5 days. Pancreatic arginine deaminase 4, an enzyme critical for NET formation, is upregulated in ischemic brain tissue. The overexpression of peptidyl arginine deiminase 4 leads to increased NET formation, accompanied by decreased vascularization and increased BBB injury. The disruption of NET by DNase 1, or the inhibition of NET formation by knockdown or the pharmacological inhibition of PAD, increases neovascularization and vascular repair and improves functional recovery. In addition, PAD inhibition reduced stroke-induced STING-mediated IFN-β production, and STING knockdown and IFN receptor-neutralizing antibody therapy reduced damage to the BBB and increased vascular plasticity. Overall, NET release was shown to impair vascular remodeling during stroke recovery (Kang et al., 2020).

Polymorphonuclear neutrophils (PMNs) have been shown to play a decisive role in post-ischemic angiogenesis and brain remodeling, possibly by promoting extracellular matrix degradation, thus enhancing the recovery process in ischemic brain tissue (Mohamud Yusuf et al., 2021).

Cellular focal death is thought to be closely related to stroke (Tang and Deng, 2018). Cellular juxtaposition is regulated by gasdermin D in the actin family of proteins, and N-gasdermin D can bind to the cell membrane and release the inflammatory factors IL-18 and IL-1β, which significantly exacerbate the inflammatory response prior to cell rupture. Gasdermin D in neutrophils mainly mediates the release of NETs, exacerbating brain injury (Long et al., 2023).

NET production has been implicated in the pathogenesis of thrombotic inflammatory states (e.g., sickle cell disease), increasing the risk of ischemic stroke (Hamam and Palaniyar, 2019). NET formation is catalyzed by the enzyme peptidyl arginine deaminase 4 and neutrophil-derived ROS, especially nicotinamide adenine dinucleotide phosphate oxidase (NOX), which interacts with peptidyl arginine deiminase 4 and thus is critical for neutrophil-driven thrombotic inflammation. Targeting peptidyl arginine deiminase 4 and NOX limits pathological H3Cit (+) neutrophils, which may further explain the attenuation of cerebral thrombosis. Overall, this study provides an effective preclinical model for the prevention and management of thromboinflammatory complications, such as ischemic stroke (Ansari et al., 2023). The investigators utilized a filament-induced transient MCAO model to induce local cerebral ischemia for 60 minutes, followed by reperfusion, and NETs were detectable at 6 hours post-stroke. Their presence increased at 12 hours, peaked at 24 hours, and then decreased again at 48 hours postischemia. Significantly, NETs were predominantly localized in the cerebral vasculature after ischemia, suggesting that NETs may play a role in secondary microthrombosis. NET formation was significantly reduced in von Willebrand factor (vWF)-deficient mice 24 hours after ischemia compared with wild-type mice, suggesting that vWF may play a role in promoting NETosis in the ischemic brain (De Wilde et al., 2023). Thus, NETs, which may act in conjunction with vWF, may be attractive targets for the development of novel therapeutic strategies (De Wilde et al., 2023).

### Neutrophil extracellular traps and thrombosis

Traditionally, thrombosis is thought to be formed by the interaction of platelets, fibrin, and red blood cells (Furie and Furie, 2008). Neutrophils serve as the first line of defense against pathogen invasion (Liew and Kubes, 2019).

The release of NETs contributes to thrombosis in acute cerebral infarction (Kimball et al., 2016; Moschonas and Tselepis, 2019; Thålin et al., 2019). Studies have shown that NETs provide a physical scaffold to capture platelets and red blood cells and also promote thrombus formation by directly activating the coagulation system. The binding of NETs with platelets and coagulation factors (such as fibrinogen and thrombin) forms a tight thrombotic core. This formation of immunothrombi is particularly prominent in ischemic stroke, where it significantly exacerbates the infarct area and neuronal damage (de Buhr et al., 2022; Knight and Kanthi, 2022).

An animal study by Wolbery et al. (2015) revealed the pathogenic roles of leukocytes, platelets, tissue factor-positive microvesicles, NETs, and factors XI and XII in thrombosis. The procoagulant activity of NETs has been reported in various thrombus-related diseases. This activity can be attributed to NETs as a scaffold for cells and a variety of coagulation factors, which stimulate fibrin deposition (Döring et al., 2017). The interaction between NETs and thrombosis plays a role in a variety of thrombus-related diseases, including stroke (Döring et al., 2017). In cerebral ischemia, neutrophils are the first cells to penetrate damaged brain tissue. They produce NETs in the brain parenchyma and blood vessels, thereby aggravating inflammation. Increasing evidence suggests that the link between NETosis and thrombosis may be one of the reasons for tissue plasminogen activator (tPA) resistance, which is a problem encountered in the treatment of stroke patients. Several DAMPs have been shown to induce NETosis and thrombosis, in which HMGB1 plays a key role. As a type of DAMP, HMGB1 can induce NETosis and thrombosis. It plays an important mediating role in thrombosis induced by NETs. HMGB1 promotes thrombosis by interacting with NETs and coagulation factors, thereby affecting the progression and treatment of stroke (Kim and Lee, 2020). Thus, understanding the role of HMGB1 in NETosis and thrombosis may provide new targets for the treatment of stroke and other thrombosis-related diseases (Kim and Lee, 2020).

An in-depth histopathologic study of a specific case of a difficult-to-remove thrombus revealed the presence of abnormally high amounts of extracellular DNA, leukocytes, vWF, and calcitonin. Extracellular DNA was reported to be positive for markers of leukocytes and the NETs, suggesting that a large amount of DNA originated from the NETs. This unique case of a stroke thrombus, which was almost resistant to thrombectomy, showed that its composition differed from the structural features of common ischemic stroke thrombi. The core of the thrombus taken is composed of extracellular DNA co-localized with vWF and microcalcifications (Staessens et al., 2021). These results support the hypothesis that vWF, NETs, and microcalcifications may promote resistance to mechanical thrombectomy.

Stroke thrombi are highly heterogeneous in composition, containing fibrin, platelets, erythrocytes, vWF, and NETs. Thrombi can be broadly divided into fibrin-rich and erythrocyte-rich thrombi. Fibrin-rich thrombi are associated with more recanalization operations, longer operative times, and poorer clinical outcomes compared to red blood cell (RBC)-rich thrombi (Jolugbo and Ariëns, 2021).

A study of thrombi from cerebral arteries removed from 21 patients with AIS reported that although distinct RBC-rich and platelet-rich zones were found, thrombi from patients with AIS contained far more platelets than RBCs, with 90% of thrombi containing less than 40% RBCs (1.5% to 37%) (Pir et al., 2022). Structurally, the erythrocyte-rich zone was relatively simple, consisting of tightly packed erythrocytes and a thin fibrin meshwork, with a paucity of nucleated cells and virtually no vWF. The platelet-rich zone was more complex than the RBC-rich zone in structure, had a thick fibrin network, and was associated with vWF. Many leukocytes were distributed in the platelet-rich zone, especially in the marginal area between the platelet-rich and erythrocyte-rich zones. The platelet-rich zone showed many activated neutrophils (myeloperoxidase^+^ and neutrophil-elastase^+^) that contained decorated DNA of citrullinated histones. Citrullinated histone-decorated DNA also accumulated extracellularly, indicating the NETosis of activated neutrophils, and NET-containing regions were associated with vWF and erythrocytes. The regions containing NETs showed a strong response to vWF, platelets, and HMGB1, suggesting a close interaction between these components (Pir et al., 2022).

Fibronectin constitutes the main matrix of the thrombus and is intertwined with DNA from neutrophil exosomes (NETs), while vWF acts as a bridge between DNA and platelets. Patient age was found to be a strong explanatory factor for the linear decrease in fibronectin relative to vWF content in thrombi, which was significant in both male AIS patients (Tóth et al., 2022). The linear regression coefficient was reduced by a factor of 3.7 in patients with AIS, and the proportion of atherosclerotic thrombi in such patients was 57%. Regardless of the anatomic location, vWF in arterial thrombi was correlated with plasma levels of inflammatory biomarkers, such as C-reactive protein, in patients with atherosclerosis, and with the abundance of local extracellular DNA. In atherothrombosis, there is a complex interaction between the vWF/NET/fibrin axis. The ratio of fibronectin to von Willebrand molecular factor in arterial thrombi correlates with plasma levels of inflammatory biomarkers, as well as with the abundance of local extracellular DNA (Tóth et al., 2022).

The study described fibrin, platelets, vWF, and NETs as the major components of the thrombus shell. Scanning electron microscopy revealed a dense, compact fibrin/platelet-rich shell and a polyhedral cell-rich core (Mereuta et al., 2023). The microfluidics study identified highly activated P-selectin-positive platelets and fibronectin forming the core, whereas secondary agonists, such as adenosine diphosphate and thromboxane, as well as loosely stacked P-selectin-negative platelets, constituted the shell. The composition, compaction, and integrity of the shell may influence the outcome of thrombolysis and revascularization (Mereuta et al., 2023).

A thrombus study of 267 patients with ischemic stroke identified the components of the thrombus, including RBCs, fibrin, vascular hemophilic factor, platelets, leukocytes, citrullinated histone 3 (a marker of the NETs), and intracellular and extracellular DNA (Vandelanotte et al., 2024). The results of the study indicated that lower RBCs, higher levels of fibrin, and increased extracellular DNA in thrombi were less likely to achieve FPR (first recanalization). These results are important guidelines for future operational or technical studies aimed at improving the recanalization rate of thrombi with fewer RBCs.

Neutrophils promote large vessel occlusion and microthrombus formation by releasing NETs, placing them at the intersection of inflammation, thrombosis, and fibrosis (Martinod et al., 2024a). Immunohistochemical staining was used to identify neutrophils, lymphocytes, macrophages, and NET patterns in thrombi formed through different mechanisms. Atherosclerotic thrombi were larger and contained more RBCs, but fewer white blood cells than cardioembolic thrombi. Athrothrombi had more lymphocytes, while cryptogenic thrombi had fewer macrophages. Notably, cardioembolic thrombi exhibited a higher expression of neutrophils and NETs than both AT and cryptogenic thrombi. The distribution of reticular NETs was significantly higher around Cardioembolic thrombi, while AT thrombi showed a mixed distribution. The differences in neutrophil and NET expression across thrombi of different etiologies may provide insight into thrombus formation mechanisms (Jabrah et al., 2024). These findings contribute to knowledge on the thrombus formation process and the potential pathological mechanisms of different types of thrombi.

### Neutrophil extracellular traps and reperfusion injury

Reperfusion therapy (such as mechanical thrombectomy or thrombolytic treatment) can restore blood flow; however, it may also activate NETs, leading to reperfusion injury. The restored blood flow brings more inflammatory cells and oxidative stress, further stimulating neutrophils to release NETs. These NETs not only exacerbate damage to the vascular endothelium and neurons but also potentially lead to secondary thrombosis, affecting the effectiveness of reperfusion therapy.

NETs are decorated with histone and granulin DNA. Recombinant DNAzyme 1 accelerated tPA-induced thrombolysis in *in vitro* experiments, whereas DNAzyme 1 alone was ineffective (van de Graaf et al., 2018). The level of NETs in thrombi may contribute to reperfusion resistance, including mechanical or pharmacologic tPA therapy, regardless of etiology. Thus, the efficacy of strategies combining DNAzyme 1 with tPA should be explored in the context of AIS.

Following ischemia/reperfusion, neutrophils accumulate in the soft meninges and perivascular space and can eventually reach infarcted brain parenchyma (Otxoa-de-Amezaga et al., 2019). Neutrophils and NETs contribute to thrombosis and resistance to reperfusion therapy(Heo et al., 2020). The histologic characterization of thrombi in stroke patients may provide some clues to the etiology of stroke, which could help identify strategies for stroke prevention. Studying thrombi may also contribute to improved reperfusion therapy, including the development of new thrombolytic drugs.

Ribonuclease kinase 2 (PKM2), a systemic regulator of inflammation, is upregulated in both humans and mice after an ischemic stroke episode. A study described the role of PKM2 in stroke pathogenesis using a murine model with prior co-morbidities (Dhanesha et al., 2022). New myeloid cell-specific PKM2-deficient (PKM2^–/–^) mice were generated, both on a wild-type background (PKM2^fl/fl^LysM^Cre+^) and a hyperlipidemic background (PKM2^fl/fl^ LysM^Cre+^Apoe^–/–^). The controls were littermate PKM2^fl/fl^LysM^Cre–^ or PKM2^fl/fl^LysMCre-Apoe^–/–^ mice. The genetic deletion of PKM2 in myeloid cells limited peripheral neutrophil inflammatory responses and reduced NETs after ischemia and reperfusion, suggesting that PKM2 promotes neutrophil hyperactivation in the stroke setting. In thin filament, autologous thrombus, and recombinant tPA models, PKM2-deficient mice with either the wild-type or hyperlipidemic backgrounds exhibited reduced infarct size and improved long-term sensorimotor recovery, regardless of sex. Laser scatter imaging showed improved regional cerebral blood flow in PKM2-deficient mice, as well as reduced post-ischemic cerebral thrombotic inflammation (intracerebral fibrinogen, platelet [CD41^+^] deposition, neutrophil infiltration, and inflammatory cytokines). The mechanistic study suggested that PKM2 regulates postischemic inflammation in peripheral neutrophils by promoting signal transducer and activator of transcription 3 phosphorylation. The nuclear translocation of PKM2 was inhibited using small molecules to improve translational significance. A significant reduction in neutrophil hyperactivation and improved short- and long-term functional recovery after stroke were found. Overall, these findings identify PKM2 as a novel therapeutic target with the promise of improving brain tissue rescue and recovery after reperfusion (Dhanesha et al., 2022).

Following ischemic stroke, large numbers of peripheral neutrophils are recruited to the region of brain injury and release NETs, leading to exacerbated BBB damage, microglia activation, and ultimately, neuronal death. The study developed a smart multifunctional delivery system to modulate immune dysregulation in the ischemic brain (Sun et al., 2023). The peptidyl arginine deiminase 4 (guanosine deiminase 4) inhibitor, Cl-amidine, was encapsulated in self-assembled liposomal nanocarriers (C-Lipo/CA) and modified with ROS-responsive polymers and fibronectin-binding peptides to enable the targeting of ischemic lesions and stimulate responsive drug release. In a mouse model of MCAO/reperfusion, C-Lipo/CA inhibited the NET release process (NETosis) and further inhibited the cGAS-STING pathway in the ischemic brain. In addition, the treatment of MCAO mice with C-Lipo/CA significantly alleviated ischemia and reperfusion injury, with the area of cerebral infarction reduced to 12.1%, compared with approximately 46.7% in the saline group. These results suggest that C-Lipo/CA demonstrates a potential therapeutic strategy to maximize mortality in ischemic stroke by integrating microglia regulation, BBB protection, and neuronal survival (Sun et al., 2023).

### Neutrophil extracellular traps and blood–brain barrier disruption

In physiological conditions, the endothelial cells lining brain microvessels are interconnected through a network of adhesion molecules and tight junction proteins, creating a selectively permeable BBB (Chou et al., 2023). Neutrophils are restricted from entering the brain parenchyma and cerebrospinal fluid(Abbott et al., 2010). However, under pathological conditions, such as infection, trauma, ischemia, and neurodegenerative diseases, neutrophils infiltrate the CNS (Cash and Theus, 2020).

Studies have shown that neutrophil-derived products, such as ROS and proteases, play a crucial role in BBB disruption. Recent observations indicate that accumulated neutrophils release NETs, which damage the BBB and directly harm the surrounding neurons (Manda-Handzlik and Demkow, 2019; Pedragosa et al., 2021; Jin et al., 2023a).

Activated astrocytes and microglia secrete pro-inflammatory cytokines that enhance the expression of adhesion molecules, such as ICAM-1, on brain microvascular endothelial cells. This upregulation promotes neutrophil adhesion (Smyth et al., 2018; Jansson et al., 2021) and facilitates the migration of immune cells into the brain parenchyma (Arts et al., 2021). Subsequently, direct interactions between neutrophils and endothelial cells, independent of transcytosis, lead to increased BBB permeability (Sienel et al., 2021). Activated neutrophils release neutrophil elastase, possibly within NETs, disrupting the adhesion junction proteins VE-cadherin and β-catenin and increasing BBB permeability. Agaphelin, a neutrophil elastase inhibitor, decreases BBB permeability in stroke, reduces infarct size, improves neurological function, and decreases mortality in mice (Leinweber et al., 2021). Histones also increase BBB permeability, particularly in the hippocampus, by disrupting adhesions and tight junctions (Villalba et al., 2020). cGAS is also activated by DNA contained in NETs and enhances type I IFN production and pro-inflammatory cytokine production (Apel et al., 2021). Thus, NETs may promote type I IFN and pro-inflammatory cytokine responses in CNS microglia, which are known to express cGAS and are involved in CNS pathology (Ding et al., 2022). NET-induced microglia activation via cGAS has been noted in a mouse model of ischemic stroke and tPA-induced cerebral hemorrhage model (Wang et al., 2021; Sun et al., 2023). A recent preprint study demonstrated that the NET-induced cGAS activation of microglia in traumatic brain injury is associated with neuroinflammation and neurological dysfunction (Turon et al., 2021). The depletion of neutrophils or the inhibition of PAD4 significantly attenuated IFN responses and ameliorated neurological deficits (Shafqat et al., 2023). Taken together, these findings suggest that NETs mediate BBB disruption, either directly through histones and various proteases or indirectly by increasing type I IFN responses.

A study identified a critical role for the TLR3-neutrophil axis in disrupting the structural-functional integrity of the BBB and distorting the developing neurovascular architecture and vascular network in children and patients with arterial ischemic stroke (Rayasam et al., 2021). Polycytidylic acid was found to enhance the neuroinflammatory milieu, promote neutrophil recruitment, significantly upregulate neutrophil elastase, and induce NETosis. The experimental confirmation of the pharmacologic inhibition of NE (sivelestat) significantly attenuated functional BBB dysfunction, NETosis, and neuroinflammation. Overall, these data reveal NE/NETosis as a novel therapeutic target for virally induced cerebral arteriopathy in children (Rayasam et al., 2021).

Activated neutrophils are thought to be involved in ischemic and infectious processes in the CNS by releasing NETs, MMP-9, and pro-inflammatory cytokines. In the context of neuropsychiatric systemic lupus erythematosus, these mechanisms promote BBB disruption, neuroinflammation, and the externalization of modified proteins on NETs that act as self-antigens (Sim et al., 2022).

A study by Huang et al. (2022) showed that edaravone dexborneol (Eda.B) significantly improved neurological function and cerebral blood flow and reduced infarct volume after experimental stroke. Eda.B downregulated NET levels in serum samples from AIS patients and cortical tissue samples from MCAO mice. Eda.B and DNase I attenuated BBB permeability by upregulating TJ-related proteins and NETs were associated with early stages of AIS. Eda.B exerted neuroprotective effects and improved BBB permeability after AIS.

Gasdermin D in neutrophils mainly mediates the release of NETs, which also exacerbates brain injury (Long et al., 2023). Under physiological conditions, NETs degrade cytokines and chemokines through proteases, exert anti-inflammatory effects, and maintain relatively stable and moderate levels. However, the excessive release of nets (NETosis), stimulated by ci/ri, will aggravate the inflammatory response and thrombosis, destroy the BBB, and trigger subsequent nerve injury and tissue damage (Luo et al., 2023).

**Figure 3:**
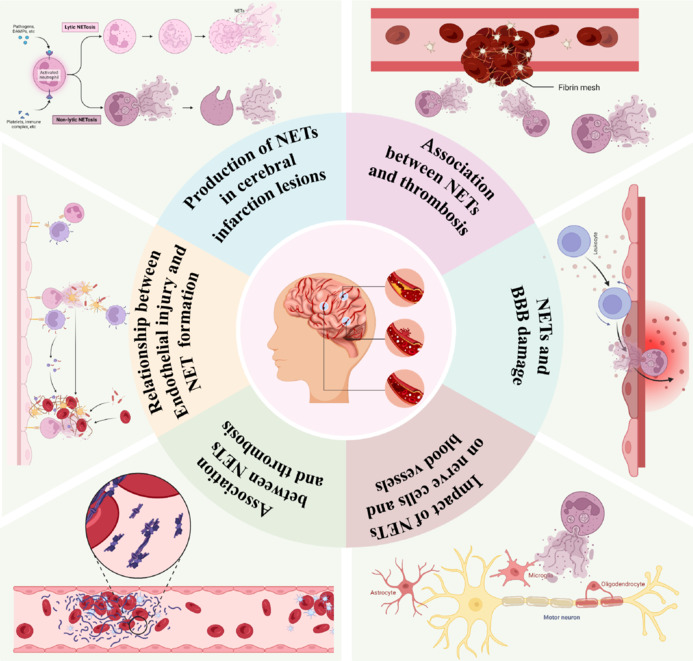
Mechanism and role of neutrophil extracellular trap (NET) formation in cerebral infarction. The figure shows the correlation between NET production and cerebral infarction. NETs cause endothelial cell damage, leading to neuronal cell injury. The relationship between NETs and thrombosis, NETs and reperfusion injury, and NETs and blood–brain barrier damage is also shown.

The inflammatory response after stroke significantly destroys the BBB and impairs its integrity. This destruction causes the infiltration of many peripheral neutrophils into the brain injury site and the release of NETs, further increasing the permeability of the BBB (Yau et al., 2022). Gamma-gc treatment effectively alleviated BBB injury, reduced neutrophil infiltration, and inhibited NET release, thereby improving ischemic injury. Transcriptome data and the subsequent validation study showed that γ-gc plays a role by activating the wnt/β-catenin pathway. Thus, γ-gc may be a promising drug for treating brain injury after ischemic stroke, improving BBB permeability, and inhibiting NET formation by regulating the wnt/β-catenin signaling pathway (Gu et al., 2024; **Figure 3**).

## Clinical Significance of Neutrophil Extracellular Traps in Cerebral Infarction

### Neutrophil extracellular traps as biomarkers of cerebral infarction

NETs play a key role in the pathological process of cerebral infarction and have been considered a potential biomarker (Qin et al., 2025). NET formation is the response of neutrophils to infection or inflammation, and their structure is composed of DNA, histones, and granulins (Li et al., 2024b). Another study by Li et al. (2024a) found that the level of NETs in the serum of patients with cerebral infarction was significantly increased, suggesting that it may participate in the inflammatory reaction mechanism, procoagulant process, and neuronal damage in the infarct lesion. Therefore, monitoring the NET levels may help to identify the occurrence of cerebral infarction early and predict its progression and prognosis.

Zhao et al. (2023b) showed that the level of NETs in serum is significantly correlated with the severity of cerebral infarction, and increases in serum NET components, such as DNA and histone levels, can be used as potential biomarkers for the early diagnosis of cerebral infarction. The presence of NETs reflects the coagulation and inflammatory status of patients with cerebral infarction, and provides a possible tool for clinical prediction and disease course monitoring.

Circulating NET biomarkers include serum or plasma peptidyl arginine deiminase 4, H3Cit, MPO, NE, nucleosomes, and DNA. Given the low specificity of a single NET biomarker, it may be more reliable to combine two or more biomarkers to reflect NET formation (Lim et al., 2020).

NETs have been shown to promote thrombosis. Laridan et al. (2017) analyzed 68 thrombi removed from patients with ischemic stroke undergoing endovascular treatment. The findings revealed that neutrophils were widely distributed in all thrombi. H3Cit (a marker for NETs) was observed in almost all thrombi. H3Cit-positive areas accounted for as much as 13.45% of the total thrombus area. The co-localization of H3Cit with extracellular DNA released from neutrophils further confirms that NETs can contribute to the formation of thrombi. The results of this study show that neutrophils are a major contributor to the formation of thrombi (Laridan et al., 2017).

A study assessed circulating NET levels by measuring plasma concentrations of double-stranded DNA and DNA-histone complexes (Lim et al., 2020). They found that circulating NET levels were increased in the initial manifestations of acute coronary syndromes and acute ischemic stroke. The findings suggest that NETs may serve as novel circulating markers for the initial diagnosis of acute coronary sydrome or AIS (Lim et al., 2020).

In a study of 148 (46.0%) patients diagnosed with severe white matter lesions (WMLs), a multiple-adjusted spline regression model showed a linear association between H3Cit levels and severe WMLs (*P* = 0.001; Zhang et al., 2023b). Increased levels of H3Cit were positively correlated with extensive WML burden in patients with ischemic stroke, suggesting that the formation of NETs may play an important role in the pathogenesis of WMLs (Zhang et al., 2023b).

β-Thromboglobulin, vWF, coagulation factor VIII, fibrinogen, activatable thrombin fibrinolysis inhibitory factor, D-dimer, and NETs have the strongest evidence for a role in ischemic stroke, making them promising markers to use in predicting conclusions about IS risk, acute-phase stroke severity, and clinical outcomes after treatment (Barakzie et al., 2023).

NET levels may serve as a valuable biomarker to predict the occurrence and functional outcome of stroke. NETs use tissue factor and platelets as scaffolds to induce thrombosis and create a procoagulant state by activating coagulation pathways and endothelial cells. Elevated NET levels in plasma and thrombi appear to be associated with increased mortality and worse functional outcomes in stroke, including AIS, intracerebral hemorrhage, and subarachnoid hemorrhage. Higher levels of NETs appear to be associated with poor outcomes after recanalization therapy and are more common in cardiogenic or cryptogenic stroke. The total neutrophil count in plasma also seems to correlate with stroke severity. NETs may be a promising predictive tool for assessing stroke severity, functional outcome, and response to recanalization therapy (Liaptsi et al., 2023).

In the early stages of AIS, higher peripheral blood white blood cell counts and neutrophil percentages, combined with lower eosinophil percentages and lymphocyte percentages (LYM%), were significantly associated with lower vWF levels in thrombi, which may indicate more severe symptoms. Therefore, the early administration of medications targeting vWF is recommended. A decrease in LYM% suggests elevated NET levels and correlates with more severe clinical symptoms. Hence, the early initiation of treatment targeting NETs should be considered (Lin et al., 2024a).

In patients with AIS, NET biomarkers in plasma are significantly elevated, and the quantity of NETs generated by neutrophils also increases. HMGB1 expression is upregulated on platelet microvesicles in AIS patients, promoting the formation of NETs. NETs enhance procoagulant activity through tissue factor and platelet activation. In the MCAO model, targeting lactadherin was shown to genetically and pharmacologically regulate the formation of NETs. Therefore, it can be concluded that platelet microvesicle-mediated HMGB1 promotes NET formation, which, in turn, exacerbates thrombosis and brain injury in AIS (Gao et al., 2024b).

A study of 106 thrombus cases by Baumann et al. (2024) tested the NET markers DNA-histone-1 complex and MPO. Free DNA (cfDNA), DNase activity, MPO-histone complexes, and cytokine panels were measured before and 7 days after thrombus extraction. The results revealed that NET markers were present in all thrombi. The median concentration of cfDNA in the blood at baseline (pre-thrombectomy) was 0.19 µg/mL, which increased to 0.30 µg/mL after 7 days. The median DNase activity at baseline was 4.33 pmol/min/mL, which increased to 4.96 pmol/min/mL after 7 days. A correlation was found between the DNA-histone-1 complex and MPO in the thrombi. The study provides evidence of an association between the number of NETs in blood and the number of NETs in cerebral thrombi with endogenous DNase activity (Baumann et al., 2024).

Ahmadi and Alimohammadi (2024) found that different types of strokes (such as atherosclerotic, cardioembolic, and cryptogenic strokes) may be associated with specific leukocyte subtypes and NET content. The presence and quantity of NETs could reflect the characteristics of thrombi and their formation mechanisms. Leukocyte subtypes and NETs, as biomarkers, have significant potential in stroke etiology research. They can provide valuable insight into the mechanisms of thrombosis formation and may be used to improve stroke diagnosis and treatment strategies.

### Correlation between serum neutrophil extracellular traps levels and cerebral infarction severity

The concentration of serum NETs is significantly correlated with the severity of cerebral infarction. By quantifying the levels of NETs, it is possible to preliminarily predict the degree of neurological impairment and prognosis in patients. Higher NET levels are closely associated with larger areas of brain tissue necrosis, more severe neurological deficits, and poorer clinical outcomes. Thus, through their mechanisms of promoting thrombosis and exacerbating BBB disruption, NETs may play a critical role in the pathological progression of cerebral infarction.

NET formation is promoted by activated platelets. In turn, NETs can also activate platelets, thereby facilitating thrombosis. NETs have been detected in both venous and arterial thrombosis. A study of 243 acute ischemic stroke patients by Vallés et al. (2017) found that NETs were significantly elevated in the plasma of these patients compared to healthy controls. NETs were increased in patients over the age of 65 years, with a history of atrial fibrillation, cardiogenic stroke, elevated blood glucose levels, and severe stroke at both admission and discharge. In multivariate analysis, elevated H3Cit (the most specific marker of NETs) was independently associated with atrial fibrillation and all-cause mortality at 1 year of follow-up, indicating that NETs play a role in the pathophysiology of stroke and are associated with severity and mortality and H3Cit may serve as a useful prognostic marker and therapeutic target for acute stroke patients.

A study of 78 AIS thrombi by Farkas Á et al. (2019) found that the DNA content in AIS thrombi was the lowest, with a DNA/fibrin ratio 2.5 times lower than that in PAD, while the H3Cit antigen was consistently present across all sites. The NETs content showed a parabolic correlation with systemic inflammatory markers and was positively correlated with patient age. The median platelet content in PAD thrombi (2.2%) was lower than that in AIS (3.9%) or coronary artery disease (3.1%), and platelet content was lower in smokers compared to non-smokers. Fibrin fibers were significantly coarser in male coronary artery disease patients (median fiber diameter 76.3 nm), while those in AIS (64.1 nm) or PAD (62.1 nm) were finer, with their diameter correlating parabolically with systemic inflammatory markers. The results indicate that the observed NET-associated thrombus structural changes reveal new determinants of thrombus stability, which ultimately affect the natural progression and treatment outcomes of patients with ischemic arterial diseases.

NETs were detected in 100% of patients with AIS and 20.8% of patients with acute myocardial infarction (Novotny et al., 2020). The abundance of NETs in thrombi was associated with poor (low) prognostic scores in patients with AIS and a reduced left ventricular ejection fraction in patients with acute myocardial infarction. The presence and levels of NETs were strongly associated with the outcomes of patients with AIS and acute myocardial infarction, emphasizing the role of NETs in thrombus stability in both diseases. Other investigators confirmed that NETs play a role in the pathophysiology of stroke and correlate with stroke severity and mortality (Zhang et al., 2023b).

Clinical data suggest that the severity of traumatic brain injury and stroke is positively correlated with the number of neutrophils in the peripheral blood and the brain injury site. In addition, neutrophil-released NETs are associated with poor prognosis in traumatic brain injury and stroke by impairing vascular regeneration and vascular remodeling. Therefore, targeting neutrophils to deliver NET inhibitors to the site of brain injury and reduce the formation of NETs may be an optimal strategy for treating traumatic brain injury and stroke. In this study by Mu et al. (2024a) an ROS-responsive neutrophil-targeted delivery system containing the peptidyl arginine deaminase 4 inhibitor GSK484 was designed and synthesized to prevent the formation of NETs at the site of brain injury. This system significantly suppressed neuroinflammation, ameliorated neurological deficits, and improved survival in traumatic brain injury and cerebral ischemia-reperfusion injury. This strategy may provide the basis for developing targeted therapeutic diagnostics for traumatic brain injury and stroke.

### Differential roles of neutrophil extracellular traps in acute and chronic phases of ischemic stroke

In the acute phase, NETs aggravate brain tissue damage through their procoagulant effect and inflammatory response and induce a larger range of neuronal necrosis. In the chronic phase, the regulatory effect of NETs may contribute to nerve repair (Papayannopoulos, 2018; Singhal and Kumar, 2022). Studies have shown that moderate regulation of the generation and clearance of NETs is expected to reduce chronic inflammatory reactions and promote nerve regeneration (Baptista de Barros Ribeiro Dourado et al., 2022; Hidalgo et al., 2022; Li et al., 2023b). Therefore, understanding the differences in the effects of NETs in different periods is crucial for optimizing treatment strategies.

#### Pathological mechanisms of acute-phase tissue injury

During the acute phase of cerebral infarction, the release of NETs is inextricably linked to the massive recruitment of neutrophils. NETs promote the formation of local thrombi and, by activating the coagulation pathway, further impede the blood supply, thereby exacerbating hypoxia and brain tissue necrosis. In addition, histones and proteases in NETs are directly toxic to neuronal cells, leading to increased brain tissue damage. The formation of NETs in this period may be an important factor in the severity of cerebral infarction.

Neutrophils can be classified into N1 and N2 phenotypes, which have been found in tumors, and similar phenotypes have been found after ischemic stroke. N1 neutrophils predominate during the acute phase of stroke (within 3 days) and cause damage to neural structures. However, the proportion of N2 neutrophils gradually increases in the later stages and exerts beneficial effects by releasing anti-inflammatory factors and other neuroprotective mediators. The N1 and N2 phenotypes are highly plastic and can be transformed into each other under certain conditions. The significant functional differences and high degree of plasticity of these phenotypes make these neutrophil subpopulations promising targets for ischemic stroke therapy (Xie et al., 2023).

In the acute phase of stroke, NETs exert detrimental effects in a platelet-TLR4-dependent manner. Thus, their pharmacological modulation could be neuroprotective. Targeting NETs during this phase represents a promising therapeutic strategy for mitigating ischemic brain injury, independent of the thrombus type (Peña-Martínez et al., 2022).

The four core histones H2A, H2B, H3, and H4, along with the linker histone H1, primarily associate with DNA to regulate gene expression within the nucleus. Under conditions of cellular activation or damage, histones can be released into the extracellular environment. Extracellular histones and histone-containing complexes, such as nucleosomes and NETs, exhibit innate immunomodulatory functions. As DAMPs, histones initiate immune responses by interacting with cell membranes and activating pattern recognition receptors, including TLR2, TLR4, and TLR9, and the receptor for RAGE. Once histones penetrate the BBB or are secreted by CNS-resident cells such as neurons, microglia, and astrocytes, they can be detected within the brain parenchyma. However, research on the DAMP-like effects of histones in CNS cells is limited. TLR4 is the only identified molecular target of extracellular histones in CNS cells, and their interactions with other PRRs expressed by brain cells require further investigation. Nevertheless, extracellular histones have been implicated in the pathogenesis of various neurological disorders associated with sterile neuroinflammation. A comprehensive exploration of the roles of these proteins and their complexes in neuroinflammatory processes may help identify novel therapeutic targets (Richards et al., 2023).

Liu et al. (2023b) have developed thrombus analogs enriched with extracellular traps (ETs) for preliminary clinical testing. The study found that ET-enriched thrombus analogs were more compact than their ET-poor counterparts. ETs were identified in both the ET-enriched thrombus analogs and patient thrombi, exhibiting morphological characteristics such as nuclear fragmentation, nuclear swelling, chromatin dispersion in the cytoplasm, intracellular and extracellular DNA/chromatin extension, and extracellular chromatin plaques and filaments. No ETs were observed in ET-poor thrombus analogs, and H3Cit expression was nearly absent. The composition and H3Cit expression levels of the ET-enriched thrombus analogs were consistent with those of patient thrombi. The results indicate that ET-enriched thrombus analogs can be consistently created *in vitro* and may assist in the preliminary development and testing of novel thrombolytic agents and thrombectomy devices.

#### Balancing the mechanisms of chronic-phase inflammation and neural repair

In the chronic phase of ischemic stroke, the role of NETs becomes more complex. On one hand, the persistence of chronic inflammation may delay the neurorepair process, leading to further deterioration of neurological function. On the other hand, a moderate inflammatory response may promote neuroregeneration and repair. Therefore, the role of NETs in the chronic phase has both potential negative impacts and possible neuroprotective effects. Studies suggest that modulating NET formation or inhibiting its harmful components could provide new therapeutic strategies for the later stages of ischemic stroke (Schauer et al., 2014; Poli and Zanoni, 2023; Wang et al., 2024a).

Most evidence from acute inflammation suggests that it is beneficial, particularly in inhibiting infections. However, in chronic sterile inflammation, such as in atherosclerosis and gallstone formation (Warnatsch et al., 2015; Jorch and Kubes, 2017; Muñoz et al., 2019), evidence indicates that the off-target effects of activated neutrophils may cause tissue damage (Muñoz et al., 2019).

**Figure 4:**
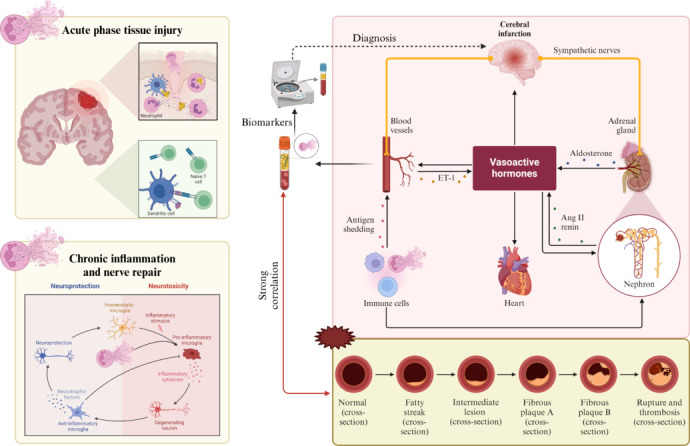
Clinical association between cerebral infarction and NETs. NETs can serve as a key biomarker for cerebral infarction and are strongly correlated with its severity. The role of NETs varies significantly across different stages of cerebral infarction. In the acute phase, NETs exacerbate tissue damage, whereas in the repair phase, NETs help alleviate tissue inflammation. Ang II: Angiotensin II; ET-1: endothelin-1; NETs: neutrophil extracellular traps.

NETs may represent a non-traditional residual risk factor for recurrent ischemic stroke, along with other contributors such as high-density lipoprotein cholesterol, triglycerides, lipoprotein-a, platelet-derived microparticles in the circulation, leukocyte-platelet complexes, the NOD-like receptor protein 3 (NLRP3) inflammasome, and monomeric C-reactive protein. Building upon the three core pillars of secondary stroke prevention—blood pressure control, lipid regulation, and antiplatelet therapy—targeting these residual risks may provide additional protection against stroke recurrence. In this context, precise identification and the quantification of residual risks related to disease heterogeneity, coupled with risk-stratified, individualized treatment strategies, hold significant potential for reducing the substantial burden of ischemic stroke (Chen and Tian, 2023; **Figure 4**).

#### Differential comparison of neutrophil extracellular traps in acute versus chronic phases of cerebral infarction

NETs demonstrate dynamically changing biological roles across different stages of cerebral infarction progression, exhibiting primarily pro-injury effects in the acute phase and pro-repair functions in the chronic phase (the specific characteristics of this temporal heterogeneity are detailed in **Additional Table 1**). From a therapeutic perspective, strategies should focus on suppressing the detrimental effects of NETs in the acute phase and balancing their reparative functions in the chronic phase. With advancing research, precision intervention strategies targeting NETs may provide novel approaches for comprehensive management throughout the entire course of cerebral infarction.

**Additional Table 1:** Temporal regulation profile of NETs in cerebral ischemia

Regulatory characteristic	Acute phase (pro-injury effects)	Chronic phase (pro-repair effects)
Key signaling pathways	HMGB1-TLR4/MyD88-NF-κB pathway activation of PAD4 (↑3.2-fold) (Kim and Lee, 2020; Zeng et al., 2022); factor XII-kallikrein system activation (Wolberg et al., 2015); NLRP3 inflammasome-mediated IL-1β release (Kluge et al., 2020; Zhou et al., 2021)	SIRTl-mediated HMGB1 deacetylation (Mu et al., 2024b); AMPK-dependent NETs clearance (Wu et al., 2023); TGF-β/Smad3 pathway promoting fibrotic repair (Muñoz et al., 2019)
Neutrophil phenotypes	Nl-dominant (MPO^+^, NE^+^) (Xie et al., 2023); release ROS and pro-inflammatory factors (TNF-α, IL-6); promote BBB disruption	Increase N2 proportion (CD206^+^) (Cai et al., 2020; Xie et al., 2023); TGF-β and IL-10; promote macrophage M2 polarization
Molecular markers	H3Cit-DNA complexes (peak at 12-24 h) (Baumann et al., 2024); elevated MPO-DNA levels (Liaptsi et al., 2023); increased plasma cfDNA	Elevate DNasel activity (↑15% at 7 d) (Baumann et al., 2024); decrease PAD4 activity (Hendrix et al., 2024); MMP-9-mediated ECM remodeling (Tang et al., 2024)
Thrombus interactions	Form dense scaffolds with vWF/fibrin (Pir et al., 2022; Toth et al., 2022); cause tPA resistance (electrostatic trapping) (Lim et al., 2020); promote microthrombosis (Martinod et al., 2024a)	Enhance fibrinolysis (uPA upregulation); reduce residual thrombotic risk (Chen and Tian, 2023); improve collateral circulation
Therapeutic ta rgets	PAD4 inhibitors (GSK484) (Zeng et al., 2022); DNase I-mediated NETs degradation (Hao et al., 2023)	Promote N2 phenotype conversion (atRA) (Cai et al., 2019); SIRT1 activators (Mu et al., 2024b); MMP-9 modulators (Tang et al., 2024)
Clinical Correlations	Infarct volume expansion (Valles et al., 2017); increased recanalization failure (Vandelanotte et al., 2024); higher DCI risk (Zeineddine et al., 2024)	Accelerate hematoma resolution (Liu et al., 2023a); improve neurological scores (mRS↓1.2 points) (Mu et al., 2024a, b); reduce recurrence risk (Chen and Tian, 2023)

The table presents a comparative overview of the dual role of NETs in the acute (pro-injury) and chronic (pro-repair) phases of cerebral ischemia. The acute phase is characterized by pro-inflammatory pathway activation (e.g., HMGB1-TLR4/NF-κB), N1-type neutrophil infiltration, and microthrombosis, which correlate with poor prognosis. In contrast, the chronic phase shifts toward anti-inflammatory repair, involving the TGF-β/Smad3 pathway, N2-type neutrophilia, and tissue remodeling to promote functional recovery. The cited studies provide an important basis for staged targeted therapy, such as inhibiting PAD4 in the acute phase and promoting N2 transformation in the chronic phase. They also provide evidence supporting each mechanism. AMPK: AMP-activated protein kinase; atRA: all-trans retinoic acid; BBB: blood-brain barrier; H3Cit: citrullinated histone H3; cfDNA: cell-free DNA; DCI: delayed cerebral ischemia; ECM: extracellular matrix; HMGB1: high mobility group box 1; IL-1β/6/10: interleukin-1β/6/10; MPO: myeloperoxidase; mRS: modified Rankin Scale; NE: neutrophil elastase; NETs: neutrophil extracellular traps; NF-κB: nuclear factor-kappa B; NLRP3: NLR family pyrin domain containing 3; OR: odds ratio; PAD4: peptidylarginine deiminase 4; ROS: reactive oxygen species; SIRT1: sirtuin 1; TGF-β: transforming growth factor beta; TLR4: Toll-like receptor 4; TNF-α: tumor necrosis factor-alpha; tPA: tissue plasminogen activator; uPA: urokinase plasminogen activator; vWF: von Willebrand factor.

## Neutrophil Extracellular Traps and Hemorrhagic Stroke

### Overview of hemorrhagic stroke and the mechanism of neutrophil extracellular trap involvement

Hemorrhagic stroke includes intracerebral hemorrhage (ICH) and subarachnoid hemorrhage (Hilkens et al., 2024). Following the onset of hemorrhagic stroke, blood vessel rupture causes a significant amount of blood to enter the brain parenchyma or cerebrospinal fluid space, triggering a strong inflammatory response. Fruh et al. (2021) showed that neutrophils are rapidly recruited to the hemorrhagic site and release NETs in response to pro-inflammatory factors.

The formation of NETs is triggered by pro-inflammatory cytokines (such as IL-1β and TNF-α), ROS, pathogen-associated molecular patterns, and DAMPs. NETs are composed of DNA, histones, and antimicrobial proteins (such as MPO and elastase) and form a mesh-like structure that captures pathogens while also creating a highly pro-inflammatory microenvironment at the hemorrhage site (Wang et al., 2024a).

### Neutrophil extracellular traps as biomarkers and therapeutic targets

NET-associated molecules (such as extracellular DNA and MPO-DNA complexes) in the plasma and cerebrospinal fluid of patients are correlated with the severity and prognosis of hemorrhagic stroke (Zhang et al., 2021c). Therefore, NETs may serve as potential biomarkers for assessing disease progression and monitoring therapeutic efficacy. Degrading NETs or inhibiting their formation, such as through DNase I-mediated degradation of extracellular DNA (Hao et al., 2023) or peptidyl arginine deiminase 4 inhibitors blocking NET formation (Zeng et al., 2022), may alleviate the inflammatory response and damage to the BBB in hemorrhagic stroke. Targeting NETs could improve neurofunctional recovery by promoting hematoma clearance, accelerating brain tissue repair, and reducing secondary injury, thereby enhancing long-term neurological outcomes.

MPO-DNA complexes, as biomarkers for NETs, have been associated with both arterial and venous thrombosis. However, their role in aneurysmal subarachnoid hemorrhage is unclear. A post-hoc analysis of a prospective observational single-center study involving 100 patients with spontaneous subarachnoid hemorrhage first detected NET biomarkers, MPO-DNA complexes, in the peripheral serum of aneurysmal subarachnoid hemorrhage patients, and found an association with delayed cerebral ischemia (DCI) (Witsch et al., 2022). Thus, a significant decrease in MPO-DNA levels may serve as an early marker of DCI, and the diagnostic potential of MPO-DNA complexes and their role as a potential therapeutic target in aneurysmal subarachnoid hemorrhage should be further explored (Witsch et al., 2022).

### Neutrophil extracellular traps and subarachnoid hemorrhage

A study confirmed that NETs were released into the ipsilateral subarachnoid space 24 hours after subarachnoid hemorrhage (Hao et al., 2023). Over time, NETs gradually increased in the brain parenchyma, expanding to the basal, cortical, and periventricular regions, with the highest concentration observed around the ventricles on day 14. In patients with aneurysmal subarachnoid hemorrhage, NETs also increased, peaking on day 7 (Weng et al., 2022). The application of RNase (bovine-derived, equivalent of human RNase 1) significantly reduced NET accumulation in the basal, cortical, and periventricular regions. The intravenous injection of RNase significantly decreased the NET load in the brain parenchyma, supporting its potential role in modulating innate immune activation following subarachnoid hemorrhage (Früh et al., 2021).

After subarachnoid hemorrhage, neutrophils are destructive and lead to adverse outcomes (Kang et al., 2020; Cai et al., 2021; Zeng et al., 2022; Witsch et al., 2024). In a mouse study, subarachnoid hemorrhage caused brain neutrophil infiltration within 24 hours, selectively inducing a pro-NETosis phenotype in the cranial neutrophils, resulting in a significant increase in iNETs on day 1, lasting at least until day 7 (Nakagawa et al., 2024). Neutrophil depletion significantly reduced iNETs, improved brain perfusion, reduced neurological deficits, and lowered the incidence of DCI (16% *vs.* 51.9%). Similarly, peptidyl arginine deiminase 4 inhibition reduced iNETs, improved neurological outcomes, and lowered the incidence of DCI (5% *vs*. 30%) in subarachnoid hemorrhage patients, whereas NET degradation slightly improved outcomes (Wong and Wagner, 2018). Aneurysmal subarachnoid hemorrhage patients with DCI exhibited elevated levels of NET markers compared to patients without DCI (Wong and Wagner, 2018). After subarachnoid hemorrhage, neutrophils from the skull were primed for NETosis, and persistent intracerebral iNETs were associated with delayed deficits (Witsch et al., 2022). The findings of this study suggest that neutrophils and NETosis could be therapeutic targets post-subarachnoid hemorrhage, as preventing vascular occlusion induced by cerebral NETs may reduce the risk of DCI. Finally, NET markers may serve as biomarkers to predict the risk of DCI development in patients with aneurysmal subarachnoid hemorrhage (Zeineddine et al., 2024).

Microthrombi play an important role in secondary brain injury following experimental subarachnoid hemorrhage; however, the specific mechanism of microthrombus formation remains unclear. Hao et al. (2023) used an intravascular puncture technique to induce subarachnoid hemorrhage in male C57BL/6 mice. They found that after subarachnoid hemorrhage, H3Cit, the marker protein for NETs, was significantly increased in the cerebral cortex and co-labeled with microthrombi. The clearance of neutrophils through anti-Ly6 antibody and DNase I treatment significantly reduced the formation of NETs and microthrombi, improving neurological deficits, brain edema, blood-brain barrier disruption, and neuronal injury 24 hours after subarachnoid hemorrhage induction. Early brain hypoperfusion following subarachnoid hemorrhage is a major determinant of poor neurological outcomes. The study also found that DNase I treatment significantly improved early cortical perfusion recovery after subarachnoid hemorrhage. Furthermore, DNase I treatment alleviated cerebrospinal fluid flow, which is related to the diffusion barrier caused by microthrombi in the perivascular space after subarachnoid hemorrhage. The association of NETs with early microthrombus formation after subarachnoid hemorrhage suggests they may be a novel therapeutic target for early brain injury (Hao et al., 2023).

Experimental studies have confirmed that NETs play an important role in the pathological process after subarachnoid hemorrhage (Wu et al., 2021; Zeng et al., 2022; Hao et al., 2023). A study of the expression dynamics of the specific marker H3Cit found that peripheral blood CitH3 levels peaked at 12 hours in the subarachnoid hemorrhage model, while brain tissue showed the highest expression at 24 hours, and its expression intensity was positively correlated with the severity of subarachnoid hemorrhage (Zeng et al., 2022). In a treatment experiment using a mouse SAH model, gsk484, a selective peptidyl arginine deiminase 4 inhibitor, significantly improved subarachnoid hemorrhage-induced brain edema and neuronal damage. In addition, DNase I treatment and neutrophil depletion experiments further verified the therapeutic value of NET inhibition. A mechanistic study showed that NETs significantly activated microglia through the TLR4/NF-κb signaling pathway, resulting in increases in the expression levels of pro-inflammatory factors TNF-α, IL-1β, and IL-6 of 2.3 times, 1.8 times, and 2.1 times, respectively. Notably, neurogenic pulmonary edema after subarachnoid hemorrhage is closely related to the excessive formation of NETs in lung tissue. Pharmacological experiments showed that gsk484 treatment reduced neutrophil infiltration in lung tissue by 42% and alveolar septal thickness by 35%. These data suggest that peptidyl arginine deiminase 4-mediated NET formation is a key link in the amplification of neuroinflammation after subarachnoid hemorrhage, and targeted intervention in this process can simultaneously improve central nervous system injury and systemic complications. This study provides an experimental basis for NETs as molecular targets for subarachnoid hemorrhage treatment (Zeng et al., 2022).

### Neutrophil extracellular traps and spontaneous intracerebral hemorrhage

Due to their procoagulant and neuroinflammatory effects, neutrophils and NETs may become interesting therapeutic targets for treating spontaneous intracerebral hemorrhage (sICH). A study by Puy et al. (2021) investigated the presence, spatial distribution, and temporal distribution of NETs in human sICH autopsy samples. Neutrophils were found in the brains of 14 patients (four males, median age: 78 years), and NETs were detected in seven of the 14 cases. Neutrophils and NETs were found not only in the hematoma but also in the surrounding tissue. The appearance of neutrophils and NETs was time-dependent and followed a biphasic pattern in the first 72 hours and between 8 and 15 days after the hemorrhage. Qualitative examination revealed that neutrophils and NETs were primarily located around dense fibrin fibers in the hematoma. These observations provide evidence of NET infiltration in the brains of sICH patients. NETs may interact with early hemostatic processes at the core of the hematoma and the surrounding neuroinflammatory response, and new perspectives for studying the role of NETs in sICH injury treatment.

### Neutrophil extracellular traps and delayed cerebral ischemia

HMGB1 is a key mediator of NET formation (NETosis) (Coleman et al., 2017). MPO-DNA complexes, as biomarkers of NETs, as well as HMGB1, are associated with DCI after aneurysmal subarachnoid hemorrhage (Coleman et al., 2017; Lin et al., 2024b). A post-hoc analysis of a prospective single-center biomarker observational study measured new markers on admission (day 0, D0) and day 4 (D4), including serum H3Cit-DNA complexes (H3Cit-DNA), peptidyl arginine deiminase 4, cell-free DNA (cf-DNA), and DNase I activity (Hendrix et al., 2024). DCI was defined as a new infarction seen on a computed tomography head scan without evidence in a follow-up scan. The study found that at D0 and D4, all serum samples showed measurable levels of H3Cit-DNA, peptidyl arginine deiminase 4, cf-DNA, and DNase I activity. No significant association was found between biomarkers on admission and the development of DCI. H3Cit-DNA levels significantly decreased from D0 to D4 in both the DCI and non-DCI groups, cf-DNA levels significantly increased, and peptidyl arginine deiminase 4 levels remained stable. In contrast, DNase I activity in the DCI group significantly decreased from D0 to D4 (*P* < 0.001), while no such change was observed in the non-DCI group (Hendrix et al., 2024). This exploratory analysis demonstrated that NET-related biomarkers, such as H3Cit-DNA, peptidyl arginine deiminase 4, cf-DNA, and DNase I activity, were present in all aneurysmal subarachnoid hemorrhage patients. A decrease in systemic DNase I activity at an early stage may increase the risk of delayed cerebral ischemia.

### Neutrophil extracellular traps and perihematomal edema

Perihematomal edema significantly exacerbates secondary brain damage in patients with cerebral hemorrhage (Chen et al., 2021), but the detailed mechanisms remain unclear. NETs are known to exacerbate neurological deficits and worsen prognosis after stroke (Kang et al., 2020; Denorme et al., 2022). A study by Tang et al. (2024) explored the potential role of NETs in the pathogenesis of cerebral edema after cerebral hemorrhage. Ly6G^+^ neutrophils surrounding hematomas were found to form NETs within 3 days after intracerebral hemorrhage. NETs reduced tight junction proteins, disrupted BBB integrity, promoted brain edema, increased neuronal apoptosis, and exacerbated neurological deficits. In contrast, the inhibition of NETs attenuated perihematomal edema, reduced neuronal apoptosis, and improved neurologic function. Mechanistically, NET-induced perihematomal edema originated from damage to the BBB tight junction via the extracellular signal-regulated kinase/matrix metallopeptidase 9 pathway coupled with the extracellular signal-regulated kinase-mediated downregulation of aquaporin-4 in the perihematomal region (Tang et al., 2024). These findings elucidate the effect of NETs on perihematomal edema, which provides promising insight into targeting NETs to alleviate cerebral edema and secondary brain injury after cerebral hemorrhage.

Taken together, the available evidence suggests that NETs play a multifaceted and critical role in the pathology of hemorrhagic stroke. From a clinical translational perspective, therapeutic strategies targeting NETs show promise. With further research, precise intervention strategies based on NETs are expected to bring new breakthroughs in the treatment of hemorrhagic stroke.

## Neutrophil Extracellular Traps, Atherosclerosis, and Other Thrombotic Cerebrovascular Diseases

### Neutrophil extracellular traps and atherosclerosis

Atherosclerosis is the result of a combination of lipid deposition in the arterial wall and chronic inflammation, which ultimately leads to vascular stenosis and thrombosis (Falk, 2006; Cheng et al., 2023). NETs play an important role in the formation and progression of atherosclerosis (Josefs et al., 2020; Adamidis et al., 2024).

NETs accelerate thrombosis in atherosclerotic plaques through their procoagulant effect (Adamidis et al., 2024). Histones, proteases, and other NET components can directly damage endothelial cells, further induce vascular endothelial dysfunction, and accelerate plaque instability (Adamidis et al., 2024). NETs can also enhance the local inflammatory response and increase the recruitment of monocytes and macrophages, thereby exacerbating the risk of plaque rupture. Therefore, NET formation is not only an important factor in the occurrence of atherosclerosis but also an important force driving arterial thrombosis (Dou et al., 2021; Zhang et al., 2025).

The formation of NETs in mouse atherosclerotic lesions was detected by Ly6G, DNA, MPO, and H3Cit staining (Yudong et al., 2018; Adamidis et al., 2024). Immunohistochemical staining revealed the colocalization of CD177, NE, and DNA in the carotid plaques of patients (Artner and Lang, 2024). Another study confirmed the presence of cluster of differentiation 66b, NE, citrullinated histone H4, and DNA at the lumen surface of vulnerable plaques, and their localization within rupture-prone plaques by immunostaining carotid and coronary artery plaques from patients (Franck et al., 2018).

Peptidyl arginine deiminase 4 is crucial for NET formation, and Shimongag et al. (2021) found that peptidyl arginine deiminase 4, as a representative of NETs, was elevated in unstable carotid plaques. The ratio of neutrophils to lymphocytes in peripheral blood is considered a biomarker for vulnerable plaques. Therefore, elucidating the role of NETs may help reveal factors that contribute to carotid plaque instability.

Carotid atherosclerotic plaque rupture and its subsequent complications are one of the leading causes of AIS. The risk of plaque rupture and subsequent thrombosis is influenced by the vulnerability characteristics of the plaque and is associated with immune system activation. A study measured the levels of NET markers, MPO-DNA complexes, in the plasma of 182 patients. Logistic regression analysis revealed a positive correlation between plaque vulnerability and plasma NET levels; however, this correlation was significant only in patients who had not used statins or anti-thrombotic drugs prior to the event (de Vries et al., 2022).

A study of carotid plaques from 26 symptomatic and eight asymptomatic patients found that the presence of NETs was associated with carotid plaque instability and lumen thrombosis, which may lead to subsequent ischemic stroke. Elucidating the role of NETs in carotid plaques may help improve the treatment of carotid artery disease (Shimonaga et al., 2022).

### Neutrophil extracellular traps and other thrombotic cerebrovascular diseases

The role of NETs in other types of thrombotic cerebrovascular diseases should not be overlooked. Cerebral venous thrombosis (CVT) is a relatively rare but severe cerebrovascular condition, typically caused by the obstruction of venous blood flow. NET levels are significantly elevated in patients with CVT, suggesting that they may have an important role in the pathologic process. Histopathological evaluation of the first CVT thrombus extracted via endovascular thrombectomy from a 47-year-old female patient revealed NETs covering 1.9% of the thrombus surface (Schwarz et al., 2024). The procoagulant function of NETs in CVT was prominent, and they directly contributed to venous thrombosis by activating the coagulation cascade reaction and enhancing fibrin deposition and formation (Jin et al., 2022). In addition, the pro-inflammatory effect of NETs in CVT aggravated ischemia and injury to local brain tissues, which further led to the deterioration of the disease (Mu et al., 2024b). A comparison of different types of thrombotic diseases would help to reveal the broad mechanisms of NET actions in cerebrovascular diseases.

The vessel wall and its endothelial lining are essential for maintaining vascular patency (Brill, 2021). When the endothelium breaks down, collagen (the first line of endothelial defense) and intravascular tissue factor (the second line of endothelial defense) are exposed to blood flow, initiating thrombosis (Silvis et al., 2017). Anticoagulant intraluminal proteins and endogenous fibrinolytic enzymes play an active role in the hemostatic process (Silvis et al., 2017). From a simplistic perspective, inflammation is one of the first responses of the immune system to infection, in which inflammation is driven by arachidonic acid-like and cytokines released by injured or infected cells (Parkin and Cohen, 2001). The cytokines that commonly regulate the inflammatory response include interleukins (primarily interleukin-6), which are responsible for communication between leukocytes, chemokines, which promote chemotaxis, and interferons, which have antiviral effects. Immunothrombosis, a mechanism of thrombosis that includes innate immune mechanisms, NETs, and immunothrombotic dysregulation, may explain some cases of deep vein thrombosis with no apparent cause, especially in elderly people, who constitute a high-risk group (Vazquez-Garza et al., 2017).

The prevalence of deep vein thrombosis symptoms after acute stroke is approximately 10%. venous thromboembolism is associated with increased hospital mortality and disability, and stroke patients have a higher incidence of hospital complications and increased 1-year mortality. After a stroke, neutrophils are among the first blood cells to respond. Importantly, the coordinated action between neutrophils, platelets, and endothelial cells contributes to the development of deep vein thrombosis. In stroke and other related immune disorders (e.g., antiphospholipid syndrome), neutrophils contribute to thrombus expansion by forming neutrophil-platelet aggregates, secreting inflammatory mediators, activating complement, releasing tissue factor, and producing NETs (Dhanesha et al., 2023).

Although the specific mechanisms of action of NETs in CVT and atherosclerosis may differ compared to cerebral infarction, their core procoagulant and pro-inflammatory functions play similar roles in these different thrombotic diseases. Thus, NETs are not only a common pathological feature of these diseases, but they may also provide a common target for the diagnosis and treatment of different thrombotic diseases (Xu et al., 2022).

A growing number of studies have shown that venous thrombosis is also related to the immune system, and inflammatory factors are recognized to be involved in venous thrombosis (von Brühl et al., 2012; Colling et al., 2021; Navarrete et al., 2023). p-Selectin causes platelet-monocyte aggregation and stimulates vascular inflammation and thrombosis (Purdy et al., 2022). Purdy et al. also reported a relationship between miRNA dysregulation and venous thrombosis, suggesting a role for miRNAs in the process of venous thrombosis (Purdy et al., 2022). Plasminogen activator inhibitor-1 (PAI-1) is a key component of the plasminogen-fibrinolytic enzyme system, and elevated levels of PAI-1, along with aging, are important risk factors for thrombosis. A recent study showed that neutrophils promote venous thrombosis by forming NETs (Huang et al., 2023).

Two animal models of CVT were used in a study by Mu et al. (2024b), simulating superior sagittal sinus thrombosis and cortical vein thrombosis. The findings showed that the cortical venous thrombosis model exhibited more severe BBB disruption and more severe cerebral hemorrhage. The reduced expression of sirtuin 1 (SIRT1) promotes the acetylation of HMGB1, leading to an increase in its intracellular translocation in the cytoplasm and its external release, which promotes the formation and recruitment of NETs. NET accumulation in the cortical brain promotes BBB damage. This establishes a vicious cycle between BBB injury and NET accumulation. Thus, the SIRT1-mediated deacetylation of HMGB1 may play a critical role in attenuating BBB damage after CVT (Mu et al., 2024b).

Takeuchi (2024) showed that pulmonary venous thrombi (PVTs) may contribute to acute myocardial infarction and ischemic stroke by releasing larger particles. In addition, PVTs can release smaller particulate matter, including NETs and their other components such as DNA and histones, to cause a variety of diseases. However, the cumulative effects of these processes need to be investigated further.

Taken together, the available studies suggest that NETs play a multifaceted pathological role in atherosclerosis and other thrombotic cerebrovascular diseases. From a clinical perspective, these findings provide important clues for the development of novel diagnostic markers and therapeutic targets. With further research, precise intervention strategies based on NETs are expected to bring breakthroughs in the prevention and treatment of thrombotic cerebrovascular diseases.

## Therapeutic Targets

**Figure 5:**
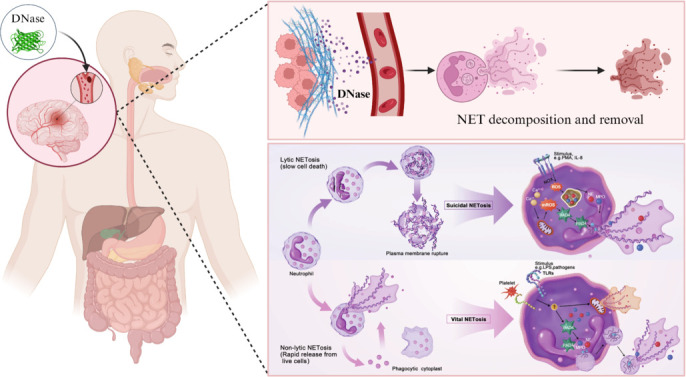
Strategies for controlling NETs. The first approach is a DNase-based method for NET degradation, followed by inhibiting the pathways involved in NET synthesis. IL-8: Interleukin-8; LPS: lipopolysaccharide; MPO: myeloperoxidase; NE: neutrophil elastase; NET: neutrophil extracellular trap; PAD4: peptidyl arginine deiminase 4; PMA: phorbol 12-myristate 13-acetate; ROS: reactive oxygen species; TLRs: Toll-like receptors.

NETs play a pro-inflammatory and pro-thrombotic role in stroke and other thrombotic diseases, making them a critical therapeutic target. Anti-inflammatory strategies that regulate the generation or degradation of NETs may effectively reduce inflammation and alleviate tissue damage in cerebral infarction (**Figure 5**).

### Pharmacological strategies for the degradation and clearance of neutrophil extracellular traps

Degrading preformed NETs is an effective approach to mitigating their pathological effects. Currently, DNase, an enzyme capable of degrading extracellular DNA, plays a crucial role in NET clearance and has demonstrated significant therapeutic potential (Du et al., 2023).

From a pathophysiological perspective, the DNA scaffold of NETs can act as an aggregation platform for coagulation factors and platelets, promoting thrombosis (Ma et al., 2024). In this process, DNase disrupts the NET structure by degrading its DNA backbone, thereby making thrombi more susceptible to lysis and reducing the risk of vascular re-occlusion (Demkow, 2023). In addition, NETs can activate pattern recognition receptors (PRRs), such as Toll-like receptors, which induce the release of pro-inflammatory cytokines (e.g., IL-1β, IL-6, and TNF-α,) and further exacerbate brain tissue injury (Wang et al., 2024d). Of interest, DNase reduces the release of these pro-inflammatory signals by degrading NETs, thereby decreasing local and neuroinflammatory responses (Wang et al., 2024d). At the microcirculatory level, NETs play an important role in microcirculatory disorders and cerebral microvascular occlusion (Nakagawa et al., 2024), and DNase treatment also improves microvascular permeability and reduces cerebral microthrombotic load, which improves perfusion in ischemic areas and contributes to neurological recovery (Nakagawa et al., 2024). Together, these findings suggest that this pharmacologic strategy offers a promising therapeutic option for future patients with cerebral infarction.

Investigators demonstrated that hemodynamic forces trigger rapid NETosis in sterile occlusive thrombi. Fibronectin inhibited NET generation, and the lack of fibronectin promoted NETs. Shear-induced NETosis was strongly negatively correlated with fibronectin in sterile occlusive thrombi (Yu and Diamond, 2019). This finding provides a new perspective for understanding the mechanism of NET formation in different thrombus types.

The levels of NETs, activated platelets, and platelet-derived particulate markers were found to be significantly higher in plasma from carotid lesion sites than in aortic blood (Zhou et al., 2020a). Of note, NETs decorated phosphatidylserine in activated platelet plasma from patients with thrombosis and ICA occlusion. Thestudy suggested that NETs carrying phosphatidylserine provide a functional platform for the deposition of platelet-derived particles and coagulation factors, thereby increasing thrombin and fibrin formation. Therapeutic intervention confirmed that DNase I and lactate dehydrogenase significantly inhibited these effects. These results strongly suggest that NETs play a key role in the hypercoagulable state of stroke patients (Zhou et al., 2020a). Further studies revealed that phosphatidylserine is not only involved in thrombus formation, but also promotes thrombus stabilization through interactions with neutrophils and platelets. In ischemic stroke thrombi, phosphatidylserine exposure may enhance the formation of NETs (Rodriguez Lucci and Schattner, 2020). Follow-up studies further validated that platelet phosphatidylserine inhibition of procoagulant platelets reduces circulating platelet-neutrophil aggregation, thereby reducing infarct volume. Platelet binding alters neutrophil function, which can exacerbate ischemic stroke-related injury, including inducing the release of NETs, which are neurotoxic and procoagulant, leading to worsened stroke outcomes. Thus, strategies to prevent the formation of NETs may offer potential therapeutic strategies for thromboembolic intervention in stroke patients (Denorme et al., 2021).

DNase I has no solubilizing effect on fibrin-rich thrombi. NETs exert deleterious effects during the acute phase of stroke in a platelet-TLR4-dependent manner, making their pharmacological modulation neuroprotective. Thus, data supported by Pena and Martinez strongly support that pharmacological modulation of NETs during the acute phase of stroke may be a promising strategy capable of repairing brain damage in ischemic disease, independent of the type of thrombus (Peña-Martínez et al., 2022).

As mentioned earlier, DNase has demonstrated some therapeutic potential for cerebral infarction in animal models and some clinical studies. However, some safety risks are associated with its long-term use. Because DNase may reduce inflammation by degrading extracellular DNA, it may interfere with the body’s immune tolerance to its own DNA, which plays an important role in autoimmune diseases such as systemic lupus erythematosus (Stabach et al., 2024). The possibility that long-term DNase use may alter the body’s tolerance to its own DNA and increase the risk of autoimmune diseases is only a theory-based speculation, and it has not been confirmed by direct experimental evidence.

In terms of other potential risks, Tong et al. (2024) showed that high concentrations of DNase may damage endothelial cells, leading to increased vascular permeability and, thus, aggravating brain edema. In addition, excessive NET degradation may affect their role in anti-infection immunity and increase the risk of infection (Wang et al., 2024a).

From the perspective of thrombus stability, beyond thrombosis promotion, NETs also contribute to thrombus stabilization and vascular repair (Zhang et al., 2025). DNase treatment may disrupt thrombus stability, thereby increasing the risk of rebleeding (Mengozzi et al., 2024). This concern is particularly relevant in patients with ischemic stroke, as rebleeding could exacerbate neurological deficits.

From a pharmacokinetic standpoint, DNase I has a relatively short half-life (typically a few hours), necessitating frequent administration to maintain therapeutic efficacy, which may impact patient adherence in clinical practice (Xia et al., 2025). Furthermore, individual variations, including genetic polymorphisms and inflammatory status, may affect DNase activity, highlighting the need for further research on personalized treatment strategies.

Current evidence demonstrates that DNase, as a key NET-degrading agent, exhibits significant therapeutic potential. However, its clinical application faces multiple challenges, including safety concerns and the optimization of dosing regimens. With advances in understanding the pathological mechanisms of NETs, DNase-based treatment strategies may offer safer and more effective therapeutic options for ischemic stroke patients.

### Molecular targets for inhibiting neutrophil extracellular trap formation

Inhibiting the formation of NETs using molecular targets is another significant approach to treating NET-related diseases. The formation of NETs relies on the activation of various signaling pathways, such as ROS and protein kinase C pathways (Tan et al., 2021). Thus, neutrophil activation and NET formation can be reduced by inhibiting these key pathways. Drugs targeting these molecular pathways, such as ROS inhibitors and protein kinase C inhibitors, were shown to effectively reduce NET formation in experimental models, thereby decreasing thrombosis and inflammation (Lu et al., 2021; Mutua and Gershwin, 2021; Zhan et al., 2023). The targeted inhibition of NETs or NET formation is emerging as a promising therapeutic strategy for ischemic thrombotic diseases.

Histone citrullination is a crucial step in the formation of NETs, primarily catalyzed by peptidyl arginine deiminase 4. Peptidyl arginine deiminase 4 is an enzyme that modifies proteins by converting positively charged arginine residues into neutral citrulline residues (Seol et al., 2025). By citrullinating arginine residues on histones H3 and H4, PAD4 induces chromatin decondensation, which serves as a mandatory prerequisite for NET formation and release (Wang et al., 2024c).

Peptidyl arginine deiminase 4 inhibitors can directly suppress its enzymatic activity. Compounds such as Cl-amidine and BB-Cl-amidine competitively inhibit peptidyl arginine deiminase 4, preventing the citrullination of arginine residues, and thereby reducing NET release (Wang et al., 2024c). Additionally, peptidyl arginine deiminase 4 inhibitors block histone citrullination, impede chromatin decondensation, and inhibit NET formation (Wang et al., 2024c). These inhibitors have been shown to reduce NET-mediated pro-inflammatory cytokine release (e.g., IL-1β and TNF-α) and decrease NET deposition in blood vessels (Van Bruggen et al., 2024), thereby alleviating NET-induced inflammation and thrombosis.

Since NETs contribute to thrombus stability and hemostasis, the excessive inhibition of NETosis may increase bleeding risk (Zhang et al., 2025). Peptidyl arginine deiminase 4 also plays a role in neutrophil-mediated antimicrobial immunity, and its inhibition may increase susceptibility to infections (Wang et al., 2024a).

Common peptidyl arginine deiminase 4 inhibitors include chloramidine, biotin-benzoyl-chloramidine, GSK484, Jiangsu Berry Institute Compound 589, thiophene dicarboxylic acid-furanyl amidine, and Yonsei University-Washington University Compound 3–56. These inhibitors reduce NET formation by suppressing peptidyl arginine deiminase 4 activity, thereby alleviating inflammation and thrombotic burden. While they have shown potential therapeutic effects in animal models and some clinical studies, further research is required to confirm their safety and efficacy (Vincent et al., 2018; Perdomo et al., 2019; Yao et al., 2022; Shi et al., 2023; Martinod et al., 2024b).

A novel ROS-responsive neutrophil-targeting delivery system has been designed to carry the peptidyl arginine deiminase 4 inhibitor GSK484, preventing NET formation at sites of brain injury. This approach significantly suppressed neuroinflammation, improved neurological deficits, and increased survival rates in traumatic brain injury and cerebral ischemia-reperfusion injury models. This strategy may serve as a foundation for targeted therapies in traumatic brain injury and stroke (Mu et al., 2024a).

A study by Seol et al. (2025) demonstrated that peptidyl arginine deiminase 4 levels increased significantly in the ischemic core and penumbra of the affected hemisphere 3–6 hours and 6–48 hours after MCAO. Strong induction of peptidyl arginine deiminase 4 was observed in neurons 12–24 hours post-MCAO. Notably, the intranasal administration of the peptidyl arginine deiminase 4 inhibitor BB-Cl-amidine at 24 hours post-MCAO significantly reduced infarct volume and improved neurological and functional outcomes, suggesting a potent neuroprotective effect of peptidyl arginine deiminase 4 inhibition in ischemic stroke. These findings indicate that peptidyl arginine deiminase 4 plays a complex role in cerebral ischemia, exhibiting neuroprotective (NETosis-independent) functions during the acute to subacute phase while exerting NETosis-inhibitory effects in later stages. This insight provides a novel perspective for the treatment of ischemic stroke (Seol et al., 2025).

HMGB-1 is a typical DAMP protein that exerts its biological functions through interactions with its receptors, including RAGE and TLR4. HMGB-1 induces the formation of NETs and promotes thrombus formation and development by interacting with platelets, NETs, and coagulation and fibrinolysis factors. Therapeutic strategies targeting HMGB-1 may provide new solutions for the management of thrombus-related diseases (Wu et al., 2018).

Sirtuin 3 (Sirt3) is a mitochondrial NAD^+^-dependent deacetylase that mitigates oxidative stress by activating superoxide dismutase 2 (Park et al., 2011). Oxidative stress contributes to enhanced arterial thrombosis (Wang et al., 2023). The study by Gaul et al. (2018) demonstrates that the loss of Sirt3 function promoted thrombosis *in vivo* by increasing extracellular trap formation and increasing tissue factor levels, suggesting that endogenous Sirt3 plays a protective anti-thrombotic role. In patients with STEMI, acute coronary thrombosis is linked to decreased expression of Sirt3 and superoxide dismutase 2 in CD14^+^ leukocytes. Therefore, NAD^+^ enhancers that activate Sirt3 may represent a novel therapeutic approach to preventing or treating thrombotic arterial occlusion in conditions such as myocardial infarction and stroke.

All-trans retinoic acid is a potent immunomodulator. Treatment with all-trans retinoic acid skews neutrophils toward the N2 phenotype, promoting their clearance by macrophages and inhibiting the formation of NETs. It demonstrates strong therapeutic effects in ischemic stroke by mitigating neuroinflammation, preventing neutrophil accumulation, promoting N2 polarization, and inhibiting NET formation. The signal transducer and activator of transcription 1 (STAT1) signaling pathway plays a decisive role in the mechanism by which all-trans retinoic acid regulates neutrophils (Cai et al., 2019).

The expression of the N2 marker CD206 remains relatively constant in neutrophils infiltrating the stroke injury site. Research indicates that the N2 phenotype, mediated by macrophages, facilitates the clearance of neutrophils and prevents further neuronal death following ischemic injury, in contrast to N0 or N1 neutrophils (Cai et al., 2020). One day after tMCAO, redirecting neutrophils toward the N2 phenotype reduced infarct volume. Additionally, conditioned medium from ischemic neurons promoted the transition of neutrophils from the protective N2 phenotype, thereby increasing extracellular trap formation. Thus, steering neutrophils toward the N2 phenotype may offer a promising therapeutic approach for ischemic stroke.

Compared with patients with normal blood glucose, hyperglycemic patients have significantly higher levels of H3Cit in their thrombi. Researchers established a permanent MCAO model in hyperglycemic mice by injecting glucose into Bio Breeding Kondo Strain-db/db and wild-type mice (Deng et al., 2020). Hyperglycemia-induced NETs were detected in the ischemic perilesional brain tissue. The inhibition of NET formation reduced infarct volume in hyperglycemic mice and alleviated neurological deficits.

The study found that ATP, acting as a DAMP, accumulates in brain tissue and induces NETosis in both brain parenchyma and circulating PMNs isolated from MCAO model mice (Kim et al., 2020b). Following MCAO, the expression of H3Cit and peptidyl arginine deiminase 4, both of which are involved in NETosis, was significantly increased in both brain parenchyma and blood PMNs. ATP or BzATP (a prototype P2X7 receptor agonist) notably enhanced the induction of peptidyl arginine deiminase 4 and H3Cit, a process dependent on P2X7R. The key mechanisms involved in ATP-P2X7R-mediated NETosis include intracellular Ca^2+^ influx, protein kinase C-α activation, and nicotinamide adenine dinucleotide phosphate oxidase-dependent ROS generation. In the MCAO animal model, treatment with apyrase, an enzyme that hydrolyzes ATP, significantly inhibited NETosis, while combined treatment with BzATP enhanced NETosis, confirming the role of ATP-P2X7R in NETosis induction. Given that ATP both induces NETosis and is expelled following NET formation, these findings suggest that ATP accumulation in ischemic brain tissue triggers NETosis and mediates the interplay between NETosis and neuronal damage, potentially exacerbating inflammation and brain injury (Kim et al., 2020b).

A study of TLR4-deficient bone marrow cell mice [TLR4(loxP/Lyz-cre)] found that neutrophils lacking TLR4 had smaller infarct volumes than control mice (Durán-Laforet et al., 2021). The absence of TLR4 maintained neutrophils in a stable juvenile state, which was partially disrupted after ischemic injury, leading to impaired normal circadian fluctuations in neutrophil activity. TLR4-deficient neutrophils showed higher phagocytic activity under baseline conditions, were more readily engulfed by microglia after stroke, and produced less ROS during the early stages of inflammation. TLR4 plays a specific role in neutrophil dynamics under both physiological conditions and tissue damage induced by stroke. The study’s findings support the idea of targeting TLR4 as a potential neuroprotective strategy, particularly in specific cell types.

A recent study has highlighted the role of the NOD-like receptor family pyrin domain-containing 3 (NLRP3) inflammasome as a key mediator in the processes of NETosis and thrombosis formation, indicating that targeting NLRP3 inhibition may represent a promising approach for reducing thrombotic events (Kumar et al., 2023). Therefore, further preclinical and clinical research is essential to explore and substantiate NLRP3 inhibition as a novel therapeutic strategy for thrombotic disorders.

A previous study observed that NETs infiltrated several brain regions using immunofluorescence and flow cytometry, including the primary motor cortex, striatum, vertical and horizontal limbs of the diagonal band, and medial septal nucleus, persisting in the brain parenchyma for up to 14 days (Li et al., 2023a). Constraint-induced movement therapy demonstrated an ability to lower NET levels and C–C motif chemokine ligands 2 and 5 in M1. Although constraint-induced movement therapy pharmacologically inhibited peptidyl arginine deiminase 4 to suppress NET formation, it did not further mitigate neurological deficits. These findings suggest that constraint-induced movement therapy may help alleviate motor dysfunction induced by cerebral ischemic injury by modulating neutrophil activation (Li et al., 2023a).

Obesity-induced hyperglycemia is a major risk factor for stroke. Integrin α9β1, expressed on neutrophils, stabilizes adhesion to the endothelium by interacting with ligands such as fibronectin containing extra domain A and fibronectin C. A study by Patel et al. (2023) revealed that stroke significantly increased neutrophil α9 expression in obese mice (*P* < 0.05 compared to lean mice). Obese mice lacking neutrophil α9 exhibited notable functional improvements over 4 weeks, with reduced infarction, enhanced cerebral blood flow, and less post-reperfusion thrombotic inflammation and NETosis (*P* < 0.05 compared with α9 (WT) obese mice). In a carotid artery thrombosis model induced by FeCl₃ injury, obese α9 (Neu-KO) mice showed reduced thrombosis susceptibility. The research identified the α9/fibronectin axis as promoting NETosis through the extracellular signal-regulated kinase pathway and peptidyl arginine deiminase 4, with neutrophil α9 worsening stroke outcomes via fibronectin-EDA, but not through fibronectin C. Anti-integrin α9 injection significantly improved functional recovery in obese WT mice, with the longest observation period being 4 weeks (*P* < 0.05 compared with controls). Deleting neutrophil-specific α9 or using pharmacological inhibitors improved long-term functional outcomes in obese hyperglycemic mice after stroke, possibly by limiting thrombotic inflammation (Patel et al., 2023).

Complement receptor 21 (CD21) is a novel phthalate-derived neuroprotectant that has shown protective effects against cerebral ischemia in rodent models. The intravenous administration of CD21 (13.79 mg/kg) significantly reduced NET components such as plasma double-stranded DNA concentration, elastase, peroxidase, neutrophil gelatinase-associated lipocalin mRNA levels, and histone H3 protein levels in ischemic brain tissue. It also decreased mRNA and protein levels of NET formation enzymes such as peptidyl arginine deiminase 4 and NET-related inflammatory mediators, including IL-1β, IL-17A, and MMP-8 and MMP-9. Additionally, CD21 demonstrated neuroprotective effects, improved cerebral blood flow, and reduced brain damage, with mechanisms including AMPK activation. As a novel phthalate derivative, CD21 protected against ischemic brain injury in rodent models by modulating the platelet-NET-thrombin axis and AMPK activation. This suggests that CD21 could be a potential drug target for treating ischemic brain injury (Wu et al., 2023; **Additional Table 2**).

**Additional Table 2:** Molecular targets for inhibiting NET formation

Molecular target	Mechanism of action	Reference
ROS, PKC	Inhibition of these key pathways reduces neutrophil activation and NET formation, thereby decreasing thrombosis and inflammation.	Tan et al., 2021
PAD4	Histone citrullination induces chromatin decondensation.	Seol et al., 2025
HMGB-1	Induce NET formation by interacting with receptors (such as RAGE and TLR4) and promoting thrombosis and the development of blood clots.	Wu et al., 2018
Sirtuin 3 (Sirt3)	Reduce oxidative stress by activating SOD2 and alleviating thrombosis; Sirt3 deficiency increases NET formation and the risk of thrombosis.	Gaul et al., 2018
All-trans retinoic acid (atRA)	Promote neutrophil polarization towards the N2 phenotype, enhance neutrophil clearance by macrophages, inhibit NET formation, and alleviate neuroinflammation and cell accumulation.	Cai et al., 2019
Hyperglycemia- induced NETs	Hyperglycemia induces NET formation; inhibiting NET formation reduces infarct size and alleviates neurological deficits.	Deng et al., 2020
ATP	Acts as a DAMP to activate P2X7R, inducing Ca^2+^ influx, PKC-a activation, and ROS production, which promote PAD4 activation and NET formation	Kim et al., 2020b
TLR4	Neutrophils lacking TLR4 exhibit higher phagocytic activity, reduce oxidative stress, inhibit NET formation, and decrease infarct size.	Durán-Laforet et al., 2021
NLRP3	Inhibition of NLRP3 inflammasome reduces NET formation, decreasing thrombotic events.	Kumar et al., 2023
CIMT	Modulate neutrophil activation, inhibit NET formation, and alleviate motor impairment caused by brain ischemia.	Li et al., 2023a
Integrin α931	Interact with ligands (Fn-EDA and fibronectin C) to promote NET formation; inhibiting α9 improves function, reduces infarction, and decreases thrombotic inflammation and NETosis.	Patel et al., 2023
CD21	Modulate the platelet-NET-thrombin axis, activate AMPK, inhibit NET formation, and improve cerebral blood flow and neuroprotection, showing protective effects against ischemic brain injury.	Wu et al., 2023

This table summarizes the key molecular targets involved in NET formation and their therapeutic potential in thrombosis and inflammation-related conditions. The listed mechanisms highlight how inhibiting these targets may reduce NETosis, thereby offering neuroprotective and anti-thrombotic benefits in diseases such as ischemic stroke. ATP: Adenosine triphosphate; atRA: all-trans retinoic acid; CD21: complement receptor 21; CIMT: constraint-induced movement therapy; HMGB1: high mobility group box 1; integrin α93l: integrin alpha-9 beta-1; NET: neutrophil extracellular trap; NLRP3: NOD-like receptor family pyrin domain-containing 3; PAD4: peptidyl arginine deiminase 4; PKC: protein kinase C; ROS: reactive oxygen species; Sirt3: sirtuin 3; TLR4: Toll-like receptor 4.

Current research demonstrates that multi-target intervention strategies for NET inhibition hold considerable clinical promise. From PAD4 inhibitors to HMGB1 antagonists, and from Sirt3 activators to integrin α9 blockers, various targeted agents effectively suppress NET formation through distinct mechanisms, thereby mitigating neuroinflammation and thrombotic burden. However, key challenges, including target specificity, optimal dosing timing, and safety profiles, must be addressed to advance clinical translation.

With a deeper understanding of the molecular mechanisms underlying NET formation, precision interventions targeting this pathological process may lead to transformative breakthroughs in the treatment of ischemic stroke and other thrombotic disorders.

### Therapeutic potential of combining neutrophil extracellular trap-targeting and anticoagulant therapy

Given the pivotal role of NETs in thrombosis, the combined application of NET-targeting therapies with anticoagulant treatments holds significant promise. Traditional anticoagulants, such as warfarin and heparin, reduce thrombus formation by inhibiting coagulation pathways. However, NET formation involves the coagulation system as well as immune and inflammatory responses. Therefore, combining anti-NET therapies with anticoagulant treatment can simultaneously inhibit multiple pathogenic pathways and improve therapeutic efficacy. Early studies have shown that this combination therapy has potential synergistic effects in reducing thrombus formation, decreasing infarct size, and improving prognosis (Fuchs et al., 2010; Chrysanthopoulou et al., 2014; Perdomo et al., 2019).

#### Synergistic and interactive effects of neutrophil extracellular traps and traditional anticoagulants

Traditional anticoagulants, such as heparin, not only have anticoagulant effects but also exhibit certain inhibitory effects on NETs. Studies have found that heparin can not only inhibit thrombin generation but also suppress the procoagulant function of NETs by binding to histones within NETs. Therefore, while exerting anticoagulant effects, heparin also modulates NETs activity, providing new perspectives for the application of traditional anticoagulants in NET-related diseases (Hogwood et al., 2020; Lelliott et al., 2020; Leberzammer and von Hundelshausen, 2023). Exploring these interactions may help optimize existing anticoagulation regimens and improve treatment outcomes for patients with cerebral infarction.

Thrombosis is associated with NETs released by neutrophils (Carminita et al., 2022), and NETs have been proposed as a mechanism of thrombolysis resistance (Zhang et al., 2021b). A study by Mengozzi et al. (2024) aimed to analyze the composition of thrombi retrieved after mechanical thrombectomy, estimate thrombus age and structure, and evaluate the relationship between thrombolytic therapy, antiplatelet drugs, and heparin use. This retrospective observational study involved 72 samples (44 from cerebral arteries and 28 from coronary arteries), which were stained with hematoxylin-eosin, neutrophil elastase antibody, and anti-histone H2B antibody, representing different components of NET formation that can be detected in the late stages of NETosis. Histochemical and digital quantitative analyses of NET content were performed. Univariate and mediation analyses found that the histological and morphological features of the samples were correlated with clinical information and pre-intervention treatments. The results showed that cerebral and coronary thrombi had different compositions, with a significantly higher proportion of lytic cerebral thrombi compared with coronary thrombi. NET expression was significantly higher in cerebral thrombi, as evidenced by increased H2B expression. Thrombolytic therapy was significantly associated with NE-positive expression, regardless of thrombus origin. After adjusting for thrombus location, antiplatelet therapy/heparin was not significantly associated with H2B/NE content. Importantly, thrombus age was the only independent predictor of NET content, with no mediating effect from thrombolytic therapy (*P* = 0.014; Mengozzi et al., 2024).

Thrombus age is the primary determinant of NET content and is associated with adverse clinical outcomes. Current treatments do not alter NET content. This study highlights the need to explore new drug additions to thrombolytic therapy to prevent NET formation or enhance their degradation, such as using recombinant human DNase I (Mengozzi et al., 2024).

#### Potential combination therapies of novel anticoagulants and anti-neutrophil extracellular trap drugs

With the continuous development of novel anticoagulants in recent years, combination therapies targeting NETs have become a research focus. For example, the combined use of new anticoagulants, such as direct thrombin inhibitors or factor Xa inhibitors, with DNase or other anti-NET drugs may further improve treatment efficacy. These new drugs have fewer side effects and better targeting capabilities, allowing more precise regulation of the coagulation system and NET formation. In the future, optimizing such combination therapies may significantly reduce thrombus formation and inflammatory damage and lower bleeding risks, bringing new hope for treating cerebral infarction and other thrombotic diseases.

Rivaroxaban is a potential oral anticoagulant. A study by Ma et al. (2017) found that rivaroxaban significantly inhibited the expression of neutrophils and NETs in deep vein thrombosis models, while also downregulating thrombin-activatable fibrinolysis inhibitor and PAI-1, ADP, plasminogen activator inhibitors, vWF, and thromboxane, as well as MPO and macrophage expression levels. Rivaroxaban protected against inferior vena cava filter thrombosis in heparin-induced deep vein thrombosis models. It also reduced the accumulation of inflammatory factors in deep vein thrombosis and venous thrombotic lesions and inhibited extracellular matrix and collagen-elastin fiber elasticity. Further research revealed that these effects of rivaroxaban are mediated through the MMP-9/NF-κB signaling pathway.

NETs play a key role in thrombosis and thrombolysis resistance, and the combined application of traditional and novel anticoagulants with anti-NET therapies shows broad prospects. Future research should further explore optimal combination strategies to improve thrombolysis, reduce inflammatory damage, and enhance clinical outcomes.

#### Synergistic effects of neutrophil extracellular traps and intravenous thrombolytic drugs

In recent years, optimizing thrombolytic therapy for acute ischemic stroke has become a research hotspot. Multiple studies suggest that targeting NETs may represent a new strategy to improve treatment efficacy (Kang et al., 2020; Denorme et al., 2022; Huang et al., 2022). Research has found that adding DNase 1 to standard t-PA therapy resulted in better *in vitro* thrombus dissolution, revealing that targeting NETs through DNase 1 may have pro-thrombolytic potential in acute ischemic stroke treatment (Laridan et al., 2017). Notably, *in vitro* r-tPA experiments showed reduced phagocytosis and oxidative bursts in granulocytes and monocytes, with decreased intracellular MPO potentially explaining reduced NETosis in patients with stroke (Vogelgesang et al., 2017). More importantly, NETs can protect thrombi from tPA degradation (Ducroux et al., 2018). This mechanism was further validated *in vitro*, in which recombinant DNase 1 accelerated tPA-induced thrombolysis, while DNase 1 alone was ineffective (van de Graaf et al., 2018).

The pathological effects of NETs are not limited to influencing thrombolysis efficacy but are also closely related to complications such as cerebral hemorrhage. NETs can cause tPA-induced cerebral hemorrhage and BBB disruption (Yinzhong et al., 2018). Considering the narrow therapeutic window for tPA administration due to damage to the BBB (Laridan et al., 2017), an important scientific question arises: whether inhibiting NETs could delay BBB disruption and extend the therapeutic window for tPA administration. This question warrants further investigation (Shafqat et al., 2023).

Animal experiments and clinical studies have provided additional evidence for NET-targeted therapy (Dzyubenko et al., 2023; Gao et al., 2024b; Mu et al., 2024a, b). The administration of DNase-I (promoting NET dissolution) can recanalize occluded vessels and improve outcomes in photothrombotic stroke, whereas tPA is ineffective. Prophylactic treatment with Cl-amidine (inhibiting NET formation) completely prevented thrombotic occlusion, with platelet TLR4 mediating NET formation after photothrombotic stroke. More clinically significant is that fresh platelet-rich thrombi obtained *in vitro* from ischemic stroke patients were effectively dissolved by DNase-I. These findings open new avenues for overcoming tPA resistance and recanalizing platelet-rich thrombi (Peña-Martínez et al., 2019).

Thrombus composition analysis further elucidates the molecular basis of combination therapy. Fibrin, vWF, and extracellular DNA from NETs all play important roles in the integrity of acute ischemic stroke thrombi. A quantitative study by Akipeddi et al. demonstrated that solutions containing DNase achieved approximately three times greater thrombolytic efficacy compared to standard tPA solutions (Akipeddi et al., 2024). Notably, DNA content was directly correlated with solubility in DNase-containing solutions, while it was inversely correlated in solutions without DNase. These data strongly indicate that the combined use of DNase and tPA has synergistic effects in dissolving stroke thrombi and suggest that DNase may serve as a potential adjunctive therapy among the currently limited thrombolytic drug options for acute ischemic stroke treatment.

Clinical challenges have prompted researchers to explore the immunological mechanisms behind ineffective reperfusion in greater depth. Recombinant tissue plasminogen activator (rtPA) is the primary recanalization therapy; however, nearly 50% of patients experience complications that lead to ineffective reperfusion. Although the specific factors causing ineffective reperfusion remain unclear, recent studies suggest that immune cells, particularly neutrophils, may influence rtPA thrombolytic efficacy through mechanisms such as NET formation (Vogelgesang et al., 2017; de La Taille et al., 2025; Huang et al., 2025). Research confirms that rtPA treatment increases neutrophil infiltration in cerebral microvessels and exacerbates damage to the BBB during ischemia (Huang et al., 2024b). Clinical observations also show that rtPA increases neutrophil counts in patients with large-area cerebral ischemia (Huang et al., 2024b). Collectively, these findings indicate that neutrophils play a key role in promoting ischemic injury and BBB disruption, suggesting they may be potential therapeutic targets (Huang et al., 2024b).

In summary, current research has revealed the multiple roles of NETs in the pathological process of acute ischemic stroke, providing a theoretical basis for developing novel combination thrombolytic strategies. Future studies should further clarify the optimal timing and methods for NET regulation to expand the therapeutic window and improve thrombolytic safety.

## Unique Perspectives, Future Research Directions, and Challenges

### Unique perspectives and key contributions

Compared with previous studies, the innovative contributions of this review are reflected in the following aspects. First, we systematically established a spatiotemporal dynamic model of NETs in stroke pathogenesis for the first time, highlighting their biphasic regulatory roles in both the acute and chronic phases, thereby providing a theoretical basis for clinical intervention timing. Second, we proposed the novel concept of a multi-dimensional NET biomarker profile, integrating indicators such as H3Cit, MPO, and DNase activity, and established specific correlations with stroke subtypes, thrombus characteristics, and clinical outcomes. Notably, we systematically compared the differential mechanisms of NETs in ischemic versus hemorrhagic stroke, elucidating their distinct pathological patterns in atherosclerotic stroke, cardioembolic stroke, cerebral venous thrombosis, subarachnoid hemorrhage, and spontaneous intracerebral hemorrhage. From a translational perspective, this review critically evaluates innovative strategies, such as neutrophil phenotype switching regulation, providing new approaches to overcome current therapeutic bottlenecks.

### Role of neutrophil extracellular traps in ischemic stroke

Although existing studies revealed the procoagulant and pro-inflammatory roles of NETs in ischemic stroke (Denorme et al., 2022; Huang et al., 2022; Li et al., 2022), the specific mechanisms remain complex and may be influenced by factors such as disease stage, individual differences, and stroke subtypes. NETs may play different physiological or pathological roles at different stages of the disease. In the acute phase, NETs could promote thrombosis, induce inflammatory responses, disrupt the BBB, and exacerbate ischemic brain injury (Denorme et al., 2022). In the chronic phase, the clearance of NETs may help reduce chronic inflammation, promote neurogenesis, and facilitate repair. However, the excessive and sustained release of NETs could impair local vascular regeneration and repair, potentially worsening neurodegenerative changes (Kang et al., 2020).

The role of NETs may vary among individuals and across different subtypes of ischemic stroke. For example, in large artery atherosclerotic stroke, NETs may be more likely to promote thrombus formation within arteries (Li et al., 2024a), whereas in cardioembolic stroke, NETs may affect embolus stability by inducing fibrin aggregation (Liaptsi et al., 2023). This underscores the need for further research into the dual role of NETs in various pathological contexts and their regulatory mechanisms.

The role of NETs in ischemic stroke is complex, and the differences in pathological stages and individual patient characteristics make NET research challenging (Gao et al., 2024b; Mu et al., 2024a, b). Future studies should focus on the dynamic role of NETs in ischemic stroke and explore molecular regulatory mechanisms to provide a basis for precise treatment and optimize therapeutic strategies targeting NETs.

### Role of neutrophil extracellular traps in hemorrhagic stroke

The mechanisms by which NETs contribute to hemorrhagic stroke, including intracerebral hemorrhage and subarachnoid hemorrhage, differ from those involved in ischemic stroke (Zeineddine et al., 2024). NETs exhibit a dual role in pathological processes, potentially exacerbating inflammation and secondary damage while also exerting protective effects in hematoma clearance and tissue repair (Hao et al., 2023; Nakagawa et al., 2024).

NETs activate macrophages and microglia, promoting the release of pro-inflammatory factors such as IL-1β and TNF-α, which exacerbate peri-hematoma edema and secondary damage (Zeineddine et al., 2024). Additionally, NETs can induce endothelial cell apoptosis, oxidative stress, and immune activation, leading to increased capillary leakage and further secondary brain injury (Puy et al., 2021). The proteolytic enzymes released by NETs can accelerate erythrocyte lysis, resulting in increased iron ion deposition and oxidative stress, which further impairs neurological function (Zhang et al., 2024). In cases of subarachnoid hemorrhage, NETs may enhance the inflammatory response in blood vessels, accelerate BBB disruption (Hao et al., 2023), and contribute to vasospasm (Nakagawa et al., 2024), thereby affecting disease prognosis.

In intracerebral hemorrhage, NETs may influence both hematoma formation and resolution. During the early phase of hematoma formation, NETs can promote coagulation responses, contributing to the stabilization of early hematomas (Liu et al., 2023a). Additionally, NETs may regulate macrophage polarization, facilitating hematoma clearance and the resolution of local edema and inflammation (An et al., 2019; Shi et al., 2023). Furthermore, NETs can influence extracellular matrix remodeling by releasing specific proteins, such as MMP-9, which play a role in tissue repair (Tang et al., 2024).

NETs play a dual role in hemorrhagic stroke (Shi et al., 2023). Current research on NETs in this context is still in its early stages, and further investigation is needed to uncover additional mechanisms. These findings could pave the way for new treatment strategies for hemorrhagic stroke and offer potential breakthroughs in optimizing therapeutic approaches.

### Technological and ethical challenges in clinical applications

Translating NET-related findings into clinical applications presents numerous technical and ethical challenges. A significant technical hurdle is the effective, rapid, and accurate measurement of NET levels and their assessment in stroke patients. Existing detection methods may lack sensitivity or specificity and can be difficult to implement on a large scale for clinical use (Masuda et al., 2017).

Therapeutic strategies targeting NETs encounter even greater challenges. For instance, in ischemic stroke, NET degradation strategies, such as the use of DNase, may help reduce thrombus load and inflammation; however, there is a potential risk of bleeding (Wang et al., 2021; Di et al., 2025). In the case of hemorrhagic stroke, the challenge is to mitigate secondary damage caused by NETs without negatively impacting coagulation function (Zhou et al., 2023). These issues require further exploration.

The long-term safety and efficacy of NET-targeted treatments, such as DNase, still need thorough evaluation (Wang et al., 2021). Special attention must be given to potential side effects in elderly patients and those with underlying chronic conditions. Ethically, it is crucial to carefully assess the potential risks and benefits to patients during experimental treatments to ensure the safety and feasibility of these therapeutic approaches.

### Need for multidisciplinary collaboration

NETs are involved in the complex pathophysiological processes of various diseases, including systemic lupus erythematosus (Garcia-Romo et al., 2011; Tsokos et al., 2016), atrial fibrillation (Arroyo et al., 2018; Mołek et al., 2023), intracerebral hemorrhage (Tan et al., 2019), cancer-associated thrombosis (Seo et al., 2019), brain edema (Vaibhav et al., 2020), peripheral arterial disease (Demyanets et al., 2020), coronary artery disease (Kluge et al., 2020; Zhou et al., 2021), acute myocardial infarction (Langseth et al., 2020; Li et al., 2024c), severe acute respiratory syndrome coronavirus 2 (Saleki et al., 2022), diabetes (Shafqat et al., 2022), disseminated intravascular coagulation (Zhang et al., 2022), myeloproliferative disorders (Xu et al., 2022), vaccine-induced immune thrombotic thrombocytopenia (Carnevale et al., 2023), neonatal hypoxic-ischemic encephalopathy (Bernis et al., 2023), COVID-19-induced multiple organ failure (Herrera et al., 2023), and left atrial enlargement (Martinod et al., 2024a). Only through interdisciplinary collaboration can we more comprehensively and systematically elucidate the role of NETs in stroke, providing stronger theoretical support for the development of future therapeutic strategies.

## Progress in Clinical Translation

In recent years, translational research on NETs as a novel target for stroke therapy has made significant progress. According to official ClinicalTrials.gov records (as of June 2025), more than 20 clinical trials targeting NETs in stroke are currently underway worldwide. One notable trial is the DNase I Phase II study (NCT05203224) led by Harvard Medical School in the United States, which is currently in the enrollment phase. This trial aims to evaluate the safety and dose-dependency of NETs in patients with acute ischemic stroke. Additionally, a study registered at Zhejiang University in China (ChiCTR2200066500) focuses on the targeted inhibition of NET formation using a nano-delivery system. This study aims to enhance the efficiency of cerebrovascular-specific drug delivery and achieve NET inhibition specific to cerebrovascular diseases.

In terms of drug development, the Food and Drug Administration has not yet approved a drug specifically indicated for ET-targeted stroke therapy, but relevant drug candidates are under development. GSK484, a PAD4 inhibitor developed by GlaxoSmithKline, holds an international patent (WO2021154941A1) and exhibits a certain degree of BBB permeability.

The landscape of intellectual property rights regarding NETs presents a three-pronged situation involving China, the U.S., and Europe. The U.S. currently leads in target patents (e.g., US20220170021A1), while China has experienced over 40% annual growth in the number of delivery system patents filed (e.g., CN114588453A) (source: WIPO Annual Report 2024). Meanwhile, the European Union continues to explore the field of NET-mediated stroke diagnosis and treatment by funding several NET-related studies through the Horizon Europe program. Notable projects include “Neutrophil Extracellular Traps Induced Glial Type I Interferon in the Pathogenesis of Tauopathy” and “Investigating the Interplay Between VWF, Platelets, and Neutrophil Extracellular Traps in Pathologies Involving Thrombosis of the Microvasculature.”

In terms of research funding, the U.S. National Institutes of Health has invested over $18 million in research on NETs related to cardiovascular and cerebrovascular diseases over the past five years. Similarly, the National Natural Science Foundation of China established a key project (No. 82330038) in 2024, focusing on exploring the role of NETs in the neural repair process following cerebral hemorrhage.

Despite several advances, the clinical translation of NET-targeted therapies still faces significant challenges. One major issue is that the intracerebral delivery efficiency of current nano-delivery systems remains below 10% (Cheng et al., 2021; Gao et al., 2024a). Additionally, in animal models, the effective time window for intervention is primarily limited to six hours after a stroke (Mutua and Gershwin, 2021; Tan et al., 2021; Denorme et al., 2022).

Looking ahead, it is essential to make advancements in multiple areas. For example, enhancing the permeability of the BBB using focused ultrasound and other technologies could significantly improve drug efficacy (Pandit et al., 2020; Rezai et al., 2024). Furthermore, establishing an early diagnostic system for NETs based on the detection of plasma-free DNA may facilitate timely intervention (Herrington et al., 2019). Finally, exploring combined immunomodulatory strategies that utilize immunosuppressive agents holds promise for achieving synergistic effects in neuroprotection and immune reprogramming (Westendorp et al., 2022).

## Limitations

Although this review covers many studies in the current field and synthesizes a wide range of evidence regarding the mechanisms and functions of NETs, several limitations should be acknowledged. The included studies primarily originate from English and Chinese databases, which may result in the omission of research published in other languages, thereby limiting the comprehensiveness of the review. Additionally, some of the cited studies are constrained by relatively small sample sizes and variations in study designs. For example, the study by Mengozzi et al. (2024) had a small sample size of 72 participants, while the research by Rodriguez Lucci and Schattner (2020) relied on preclinical models, which may affect the generalizability of their conclusions and the stability of their results. Furthermore, due to the lack of long-term follow-up data, it is currently impossible to make an accurate assessment of the long-term effects of certain interventions. While these preliminary results provide valuable mechanistic insights, their broader clinical applicability requires validation through large-scale, multicenter standardized protocols and enhanced access to multilingual databases to improve the reliability and generalizability of the findings. Long-term follow-up studies are also recommended to better assess the lasting effects of the interventions.

## Conclusion

NETs play a dual role in stroke, exerting significant and complex effects on pathophysiological processes. They function as both pathological promoting factors and potential therapeutic targets in stroke. While current research has highlighted the potential of NETs as a therapeutic target, further exploration is necessary to fully understand their mechanisms of action and how to effectively intervene. Developing specific therapeutic strategies aimed at targeting NETs may offer more precise and effective solutions for treating stroke and other thrombotic diseases.

## Additional files:

***Additional Table 1:*** *Temporal regulation profile of NETs in cerebral ischemia.*

***Additional Table 2:*** *Molecular targets for inhibiting NET formation.*

## Data Availability

*All relevant data are within the paper and its Additional files*.
